# Continent‐Wide Drivers of Spatial Synchrony in Breeding Demographic Structure Across Wild Great Tit Populations

**DOI:** 10.1111/ele.70079

**Published:** 2025-02-18

**Authors:** Joe P. Woodman, Stefan J. G. Vriend, Frank Adriaensen, Elena Álvarez, Alexander Artemyev, Emilio Barba, Malcolm D. Burgess, Samuel P. Caro, Laure Cauchard, Anne Charmantier, Ella F. Cole, Niels Dingemanse, Blandine Doligez, Tapio Eeva, Simon R. Evans, Arnaud Grégoire, Marcel Lambrechts, Agu Leivits, András Liker, Erik Matthysen, Markku Orell, John S. Park, Seppo Rytkönen, Juan Carlos Senar, Gábor Seress, Marta Szulkin, Kees van Oers, Emma Vatka, Marcel E. Visser, Josh A. Firth, Ben C. Sheldon

**Affiliations:** ^1^ Edward Grey Institute of Field Ornithology, Department of Biology University of Oxford Oxford UK; ^2^ Department of Animal Ecology Netherlands Institute of Ecology (NIOO‐KNAW) Wageningen the Netherlands; ^3^ Evolutionary Ecology Group, Department of Biology University of Antwerp Antwerp Belgium; ^4^ ‘Cavanilles’ Institute of Biodiversity and Evolutionary Biology University of Valencia Paterna Spain; ^5^ Institute of Biology, Karelian Research Centre Russian Academy of Sciences Petrozavodsk Russia; ^6^ Centre for Research in Animal Behaviour University of Exeter Exeter Devon UK; ^7^ Centre d'Ecologie Fonctionnelle et Evolutive Univ Montpellier, CNRS, EPHE, IRD Montpellier France; ^8^ CNRS, Department of Biometry and Evolutionary Biology (LBBE) University of Lyon, University Lyon 1 Villeurbanne France; ^9^ Anthropogenic Effects Research Group Swiss Ornithological Institute Sempach Switzerland; ^10^ Behavioural Ecology, Department of Biology Ludwig Maximilians University of Munich Martinsried Germany; ^11^ Department of Ecology and Genetics, Evolutionary Biology Centre Uppsala University Uppsala Sweden; ^12^ Department of Biology University of Turku Turku Finland; ^13^ Centre for Ecology and Conservation University of Exeter Penryn UK; ^14^ Department of Wildlife Environmental Board Pärnu Estonia; ^15^ Behavioural Ecology Research Group, Center for Natural Sciences University of Pannonia Veszprém Hungary; ^16^ HUN‐REN‐Prince Edward Island Evolutionary Ecology Research Group University of Pannonia Veszprém Hungary; ^17^ Ecology and Genetics Research Unit, Faculty of Science University of Oulu Oulu Finland; ^18^ Museu Ciències Naturals Barcelona Spain; ^19^ Institute of Evolutionary Biology, Faculty of Biology, Biological and Chemical Research Centre University of Warsaw Warsaw Poland; ^20^ Behavioural Ecology Group Wageningen University & Research (WUR) Wageningen the Netherlands; ^21^ Research Programme in Organismal and Evolutionary Biology, Faculty of Biological and Environmental Sciences University of Helsinki Helsinki Finland; ^22^ School of Biology University of Leeds Leeds UK

**Keywords:** age structure, demographic structure, great tit, mast seeding, Moran effect, *Parus major*, population dynamics, spatial synchrony

## Abstract

Variation in age structure influences population dynamics, yet we have limited understanding of the spatial scale at which its fluctuations are synchronised between populations. Using 32 great tit populations, spanning 4° W–33° E and 35°–65° N involving > 130,000 birds across 67 years, we quantify spatial synchrony in breeding demographic structure (subadult vs. adult breeders) and its drivers. We show that larger clutch sizes, colder winters, and larger beech crops lead to younger populations. We report distance‐dependent synchrony of demographic structure, maintained at approximately 650 km. Despite covariation with demographic structure, we do not find evidence for environmental variables influencing the scale of synchrony, except for beech masting. We suggest that local ecological and density‐dependent dynamics impact how environmental variation interacts with demographic structure, influencing estimates of the environment's effect on synchrony. Our analyses demonstrate the operation of synchrony in demographic structure over large scales, with implications for age‐dependent demography in populations.

## Graphical abstract

We assess the spatial synchrony of breeding demographic structure across 32 great tit populations over a 67‐year period, covering > 130,000 birds over 3200 km. We find that larger average clutch sizes, colder winters, and greater beech crops are associated with younger populations, and that demographic structure remains synchronous over distances up to 650 km. However, environmental and reproductive variables do not significantly affect the scale of this synchrony, except for a moderate influence of beech masting.

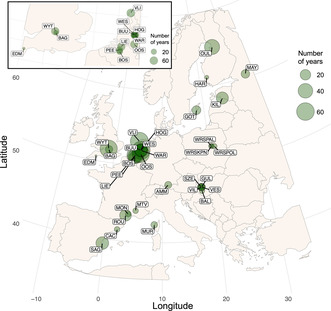

## 1 Introduction

Age‐specificity in individual‐level traits means that variation in population age structure can feed through to affect various population processes. For example, variation in age structure can influence population‐level social functioning (Siracusa et al. 2023; Woodman et al. 2024) and population growth rate (Caswell 2000; Sibly and Hone 2002). Further, the influence of age structure on population vital rates is self‐reinforcing, in that when demographic rates change, this affects the number of individuals that are recruited and die, thus shaping the overall distribution of age across a population. For example, a population's age structure may become younger either through mortality among older individuals, or when recruitment is greater. As such, variation in age structure arises when demographic rates vary, which may be influenced by environmental variability affecting recruitment and age‐specific mortality (Koons et al. 2016; Rollinson et al. 2021). A considerable amount of research has identified within‐population temporal variation in age structure (Coulson et al. 2004; Coulson, Gaillard, and Festa‐Bianchet 2005; Gamelon et al. 2016), yet relatively little is known about the spatial scale at which age structure varies, whether temporal dynamics differ between populations, and what between‐population differences in fluctuations in age structure suggest about the drivers of its variation.

Spatial synchrony is the concurrent change in time‐varying characteristics of spatially‐distinct populations (Bjørnstad, Ims, and Lambin, 1999; Liebhold, Koenig, and Bjørnstad 2004), which operates across many animal populations (Elton 1924; Moran 1953; Wan et al. 2022). Spatial synchrony can increase population stability (Paradis 1997; Ruxton 1994), but highly synchronous dynamics may impose risk of species extinction if population crashes occur simultaneously (Heino et al. 1997). Research has identified spatial synchrony in survival (Olmos et al. 2020), body mass (Herfindal et al. 2020), breeding success (Olin et al. 2020; Vriend et al. 2023), phenology (Vriend et al. 2023), and population size (Bjørnstad, Stenseth, and Saitoh, 1999; Hansen et al. 2020; Koenig 1999), particularly in birds (Mortelliti et al. 2015; Paradis et al. 1999, 2000; Sæther et al. 2007). However, despite the interrelated dynamics between population growth and age structure, little research has assessed spatial synchrony of age structure and the mechanisms that might drive this.

Spatial synchrony arises from three primary mechanisms: dispersal between populations (Kendall et al. 2000; Ripa 2000); interspecific trophic interactions with other organisms that are spatially synchronised (Ims and Andreassen 2000; Jones, Doran, and Holmes 2003; Selås 1997); or a common influence on populations from environmental variables that are correlated in space—the “Moran effect” (Moran 1953; Ranta et al. 1997). Quantifying the spatial scale at which age structure co‐fluctuates and whether any of the above mechanisms drive its spatial synchrony will advance understanding of fundamental concepts in the ecology of how populations are structured. Further, gaining insight of spatial synchrony in age structure due to environmental regulation is relevant for understanding the effects of climate change on population dynamics, particularly considering that highly synchronous dynamics might induce simultaneous population crashes and prevent the possibility of demographic rescue (Engen, Lande, and Sæther 2002; Mills 2012).

In this study, we assess spatial synchrony of variation in demographic structure across 32 European great tit breeding populations. We first assess whether fluctuations in the proportion of subadult breeders are explained by reproductive and environmental factors that vary at different spatial scales. Second, we quantify whether temporal fluctuations in this demographic structure depend on distance between populations, and whether such spatial synchrony is explained by variation in reproductive and environmental variables. By assessing the influence of separate explanatory variables, we identify how aspects of reproductive and environmental variability differentially influence variation in demographic structure, and their role in synchronising breeding population dynamics.

## 2 Methods

### 2.1 Study Systems and Data Collection

**FIGURE 1:**
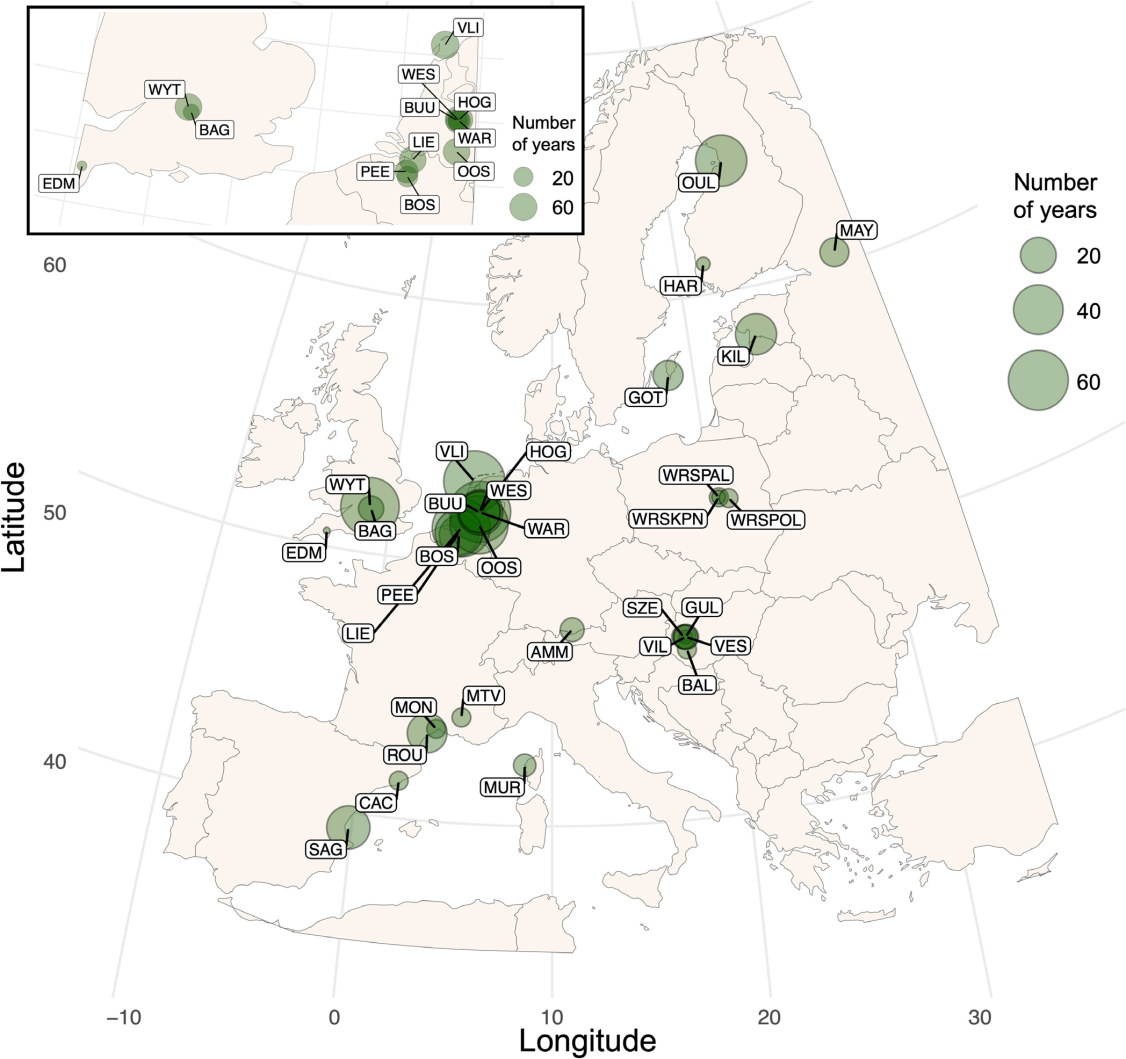
Map of the 32 great tit breeding populations across Europe, with point size relative to the number of years in the time series. The box in the top left shows the populations in The United Kingdom, Belgium, and The Netherlands in closer detail. Information for each study population can be found in Table S1.

The great tit *Parus major* is a passerine bird found in mixed woodlands across much of the Western Palearctic. Their reproductive lifespan ranges from 1 to 9, averaging 1.8 years (Bouwhuis et al. 2009; Woodman et al. 2022). Although there are some continuous changes with age (Bouwhuis et al. 2009), the main age effects on individual‐level traits and population processes are captured by two age‐classes: 1‐year‐olds (hereafter subadults) and older (hereafter adults, Gosler 1993; Harvey et al. 1979; Perrins 1979; Gamelon et al. 2016, 2019; Woodman et al. 2022). Great tits generally undertake one breeding attempt during a single annual breeding season April–June (in some parts of their range second clutches can occur, Verhulst 1998; Visser et al. 2003). Data used here are from 32 populations (Figure 1), the geographical range of which represents a large part of the species' breeding range (Sullivan et al. 2009). Generally, data collection at these sites included regular visits to nest‐boxes during breeding to track reproductive attempts, individually mark chicks and breeding individuals, and record their morphometrics, sex and age. Age is based either on year of hatching for local birds, or plumage characteristics for immigrants, where subadults and adults are discriminated based on feather moult (Svensson 1992). Further details of data collection and metadata for populations can be found through the Studies of Populations of Individual Birds (www.spibirds.org, Culina et al. 2021) and the Supporting Information S1.

### 2.2 Breeding Demographic Structure

We assigned age to all breeding great tits with known identity, across which exact year of hatching (birds first marked as chicks or subadults) was known for 82.5% of 135,967 captures. Birds first captured as adults (15.3% and 20.1% of breeding females and males, respectively) were assigned an age of 2, with subsequent age estimates based on this. Given annual mortality rates > 50% this assumption is likely to be accurate in most cases (Bouwhuis et al. 2009) – also, in these cases, individuals are still accurately designated as ‘subadult’ or ‘adult’. In total, age was assigned to 62.1% of parents where at least one egg was laid (due to some studies' protocols not always including parental identification, nests failing prior to capture, and unsuccessful trapping attempts, parental identity was unknown in some cases).

For each year, we calculated the proportion of each breeding population consisting of subadults. While this is a univariate ratio that describes breeding demographic structure without fully capturing the multivariate nature of age structure, it is a relevant proxy for age structure and provides an interpretable measure of the relative abundance of young to old individuals. Moreover, the proportion of subadults has previously been shown to be important for population processes including density regulation and population‐level breeding behaviour in this species (Gamelon et al. 2016, 2019; Woodman et al. 2022). However, we also considered alternative aspects of demographic structure by calculating five additional descriptors (mean population age; proportion of senescent individuals; and change in the three population demographic structure descriptors compared to a running mean, Supporting Information S1). We calculated these for every annual population, but only used data from years where the population included at least 20 individuals (mean, IQR: 230, 60–356) and > 25% of the population was aged (mean, IQR: 56.0%, 36.1%–78.2%, Supporting Information S1). In total, the study spanned 1956–2022, comprising 702 study years and 131,150 captures of 77,964 breeding individuals.

### 2.3 Reproductive and Environmental Variables

We assessed how reproductive and environmental variables that vary at different spatial scales relate to breeding demographic structure. First, we considered the influence of within‐population average clutch size in year t−1 on demographic structure in year t. We would expect variation in mean clutch size to affect the demographic structure of the following breeding season, where large average clutch sizes would lead to more recruits (Ahola et al. 2009) and therefore a higher proportion of breeding subadults the next year, thus we test this prediction here. We calculated within‐population average clutch size as the mean number of eggs produced per breeding attempt within a breeding season.

Second, we considered two climatic variables: temperature and precipitation. We calculated the average mean daily temperature (°C) and the average daily precipitation sum (mm) from the E‐OBS dataset (Cornes et al. 2018) across four periods preceding the focal breeding season for each population: June–August (hereafter summer); September–November (autumn); December–February (winter); and March–May (spring). We also considered the frequency of extreme climatic events (ECEs) by calculating the number of ‘cold ECEs’ and ‘hot ECEs’ June–May. We define ECEs as events with an observed occurrence in the extreme 5% of the tail of the relevant distribution across the entire study period (1956–2022) in each population separately (Marrot, Garant, and Charmantier 2017; Moreno and Møller 2011). Thus, a cold ECE occurred when minimum daily temperature was less than the 5% threshold; and a hot ECE occurred when maximum daily temperature exceeded the 95% threshold.

Third, we considered European beech *Fagus sylvatica* masting, an environmental variable which is generally understood to vary at a larger spatial scale than variation in temperature and precipitation. Beech masting is the annual production of seeds (Kelly 1994), which constitute part of the winter diet of great tits, thus influencing survival, particularly in the first‐year of life (Perdeck, Visser, and van Balen 2000). The distribution of beech does not cover the entire range of populations assessed here, and in southern Europe is restricted to higher altitudes (Bolte, Czajkowski, and Kompa 2007). However, masting‐related demographic fluctuations in tits are synchronised across regions with and without beech, suggesting that beech masting is correlated with fruiting of other tree species, such that years with a large beech crop are rich in other food resources, promoting survival across different habitats (Klomp 1980; Perrins 1966). Thus, for each annual population, we obtained a masting value from a continental‐scale dataset of beech masting up to 2017 (MASTREE+, Hacket‐Pain et al. 2022), using the masting value from a data collection site closest to that of each population in the year preceding breeding. The central coordinates for all sites were less than 1500 km from the focal breeding population, which is the spatial scale at which masting remains synchronised (Bogdziewicz et al. 2021), and most were much closer (median, IQR: 143 km, 88–297 km). To assess the influence of masting at a more local scale, we created a subset of populations within the distribution of beech (Figure S1) and where data was collected within 100 km of the population (12 populations, *n* = 188 population‐years; further details for reproductive and environmental variables in Supporting Information S1).

### 2.4 Variation in Breeding Demographic Structure and Explanatory Variables

First, we investigated the effects of the reproductive and environmental variables on breeding demographic structure. For each explanatory variable we constructed a linear mixed‐effects model of the form.

(1) $$y_{i,j}=\beta_{\mathrm{int}}+u_{\mathrm{int},i}+\left(\beta_{\text{expl}}+u_{\text{expl},i}\right)Z_{i,j}+\varepsilon_{i,j}$$

where y is the normalised subadult proportion per breeding population i and year j, $\beta_{\mathrm{int}}$ is an intercept, $u_{\mathrm{int},i}$ denotes random intercepts for each population assumed to have a normal prior distribution with mean 0 and standard deviation $\sigma_{u_{\mathrm{int}}}$, $\beta_{\text{expl}}$ is a slope for the explanatory variable, $u_{\text{expl},i}$ denotes random slopes for the explanatory variable for each population assumed to have a normal prior distribution, $Z_{\mathrm{ij}}$ is the normalised explanatory variable for each annual population, and $\varepsilon_{\mathrm{ij}}$ is the residual error, assumed to have a normal prior distribution. This model was run for the 13 explanatory variables separately, as many of the environmental variables are highly correlated, thus leading to multicollinearity issues and making interpretation of individual effects challenging.

These models were run using *brms* version 2.18.0 (Bürkner 2017). We used default priors and ran four Markov chains for 6000 iterations with a burn‐in of 3000, resulting in 12,000 posterior samples. Chain convergence was evaluated using the diagnostic $\hat{R}$ and effective sample size (Vehtari et al. 2021). We also ran the same models using alternative age structure descriptors (Supporting Information S1). The explanatory variables and demographic structure descriptors were z‐normalised such that their relative effects could be assessed.

### 2.5 Spatial Synchrony of Variation in Breeding Demographic Structure

Second, we analysed whether breeding demographic structure fluctuations are spatially synchronous, and whether this is explained by variation in the reproductive and environmental variables. Following Engen et al. (2005), we calculated a spatial autocorrelation function of the form.

(2) $$\rho \left(d\right)=\rho_{\infty }+\left(\rho_{0}-\rho_{\infty }\right)e^{-d^{2}/2l^{2}}$$

where the synchrony estimate $\rho \left(d\right)$ resembles a Pearson correlation that quantifies the degree of synchrony in breeding demographic structure fluctuations as a function of distance. $\rho_{0}$ and $\rho_{\infty }$ are correlations of demographic structure as distance approaches zero and infinity, respectively; $e^{-d^{2}/2l^{2}}$ is a Gaussian positive definite autocorrelation function where d is distance between populations (in kilometres), and l is the standard deviation representing a standardised measure of the scale of spatial autocorrelation, i.e. the characteristic scale at which temporal variation of an ecological property remains correlated (Jarillo et al. 2018; Lande, Engen, and Sæther 1999). The model assumes that the spatial autocorrelation structure is Gaussian such that the parameters $\rho_{0}$, $\rho_{\infty }$ and l are positive (as described in Engen, Lande, and Sæther 2002; Lande, Engen, and Sæther 1999). While it is possible that correlation in demographic structure between any two populations is negative, here we assume that correlation cannot be below zero on average. Modelling negative correlations on average is possible through non‐parametric approaches (e.g., Bjørnstad, Stenseth, and Saitoh, 1999), but using the parametric approach applied here is beneficial when assessing wide‐scale spatial synchrony in ecological variables (Bjørnstad, Ims, and Lambin, 1999; Engen et al. 2005; Herfindal et al. 2020; Vriend et al. 2023). Specifically, this approach allows for formal comparisons of synchrony in demographic characteristics by providing estimated parameters, such as the standard deviation (l), which non‐parametric methods do not yield (Lande, Engen, and Sæther 1999; Grøtan et al. 2005). However, we also estimated synchrony using a semi‐parametric approach to compare our main results with those obtained from a method allowing for on average negative synchrony (Supporting Information S1).

The normalised demographic structure variables of all populations in each year were assumed to have a multivariate normal distribution where ${\tilde{y}}_{t}\sim \mathrm{MVN}\left(0,\Sigma_{t}\right)$. The diagonal elements of the variance–covariance matrix were set to 1, and the off‐diagonal elements were defined by $\rho_{0}$, $\rho_{\infty }$ and l given distance d between populations. The fitting of the autocorrelation function to the data and estimation of parameters were performed using maximum likelihood estimation. Data from different populations were collected over a variable number of years, thus time series that overlap for longer were given more weight in the likelihood calculation in direct proportion to the number of overlapping years (Engen et al. 2005). Overall log‐likelihood was the sum of annual log‐likelihoods optimised numerically to provide estimates for $\rho_{0}$, $\rho_{\infty }$ and l. As stated previously, this means that while individual correlations between populations can be negative, the model's best‐fit parameters reflect an average positive trend in synchrony. The distributions of these spatial synchrony parameters were obtained by parametric bootstrapping involving simulation from the multivariate normal distribution, based on the yearly set of populations in the data and the estimated spatial synchrony parameters (Engen et al. 2005; Lillegård, Engen, and Sæther 2005). This was done 2000 times, resulting in 2000 bootstrap replicates. The multivariate normal distribution was constructed using *mvtnorm* version 1.1–3 (Genz et al. 2021). Given that some research suggests spatial synchrony has increased over time in natural populations (Koenig and Liebhold 2016), we additionally ran our spatial autocorrelation model on a subset of the data (2000–2022) to assess whether there was greater synchrony in more recent years.

Finally, we assessed the influence of reproductive and environmental variables in explaining spatial synchrony of breeding demographic structure. Following previous methods (e.g., Grøtan et al. 2005; Sæther et al. 2007; Vriend et al. 2023), the proportion of subadults in each annual breeding population was regressed against population‐specific explanatory variables in separate linear models. The residuals from these were then normalised and used as the variable of interest in the spatial synchrony model (Equation (2)). This allowed us to calculate synchrony in demographic structure once the effects of explanatory variables have been accounted for, based on differences in distance‐decay patterns. While some work highlights that when multiple environmental variables act simultaneously, it can be difficult to discern the effect of any one on spatial synchrony (Abbott 2007; Reuman et al. 2023), the approach employed here is useful as it provides estimated parameters allowing for formal comparison of spatial synchrony with and without accounting for environmental variability. All analysis was run in R statistical software version 4.2.2 (R Core Team 2021).

## 3 Results

### 3.1 Variation in Breeding Demographic Structure and Explanatory Variables

**FIGURE 2:**
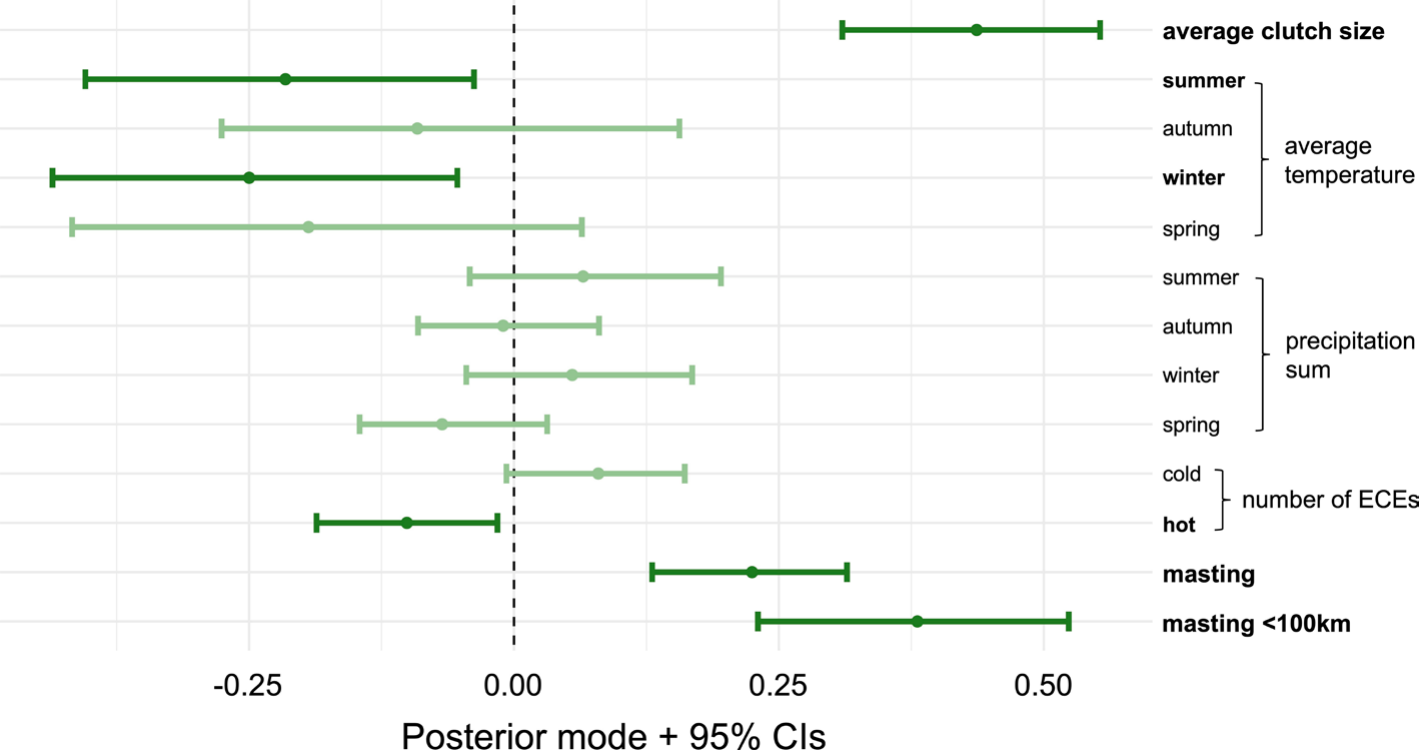
Posterior modes obtained from linear mixed‐effects models which analyse the association between temporal variation in the proportion of populations consisting of subadults and 13 reproductive and environmental variables across 32 great tit breeding populations. Each point represents the fixed‐effect slope ($\beta_{\text{expl}}$ in Equation (1)) for a specific predictor variable (on the y‐axis), and error bars denote 95% credible intervals. Points and error bars are reduced in saturation when credible intervals overlap zero, and explanatory variable text is bolded when they do not.

We found marked temporal variation in the proportion of annual breeding populations consisting of subadults, which ranged 0–0.89 across the 32 populations over 1956–2022. We found that increased average clutch sizes were associated with a larger proportion of breeding subadults the following year (Figure 2; Table S2). We also found that variation in breeding demographic structure related to climatic factors, where breeding populations with a smaller proportion of subadults followed warmer summers and years with more frequent hot ECEs. However, we found no effect of precipitation. Breeding populations had higher proportions of subadults in years following winters with a large beech crop, and this positive relationship was stronger when only assessing populations within 100 km of beech data collection (Figure 2; Table S2 for all results).

### 3.2 Spatial Synchrony of Variation in Breeding Demographic Structure

**FIGURE 3:**
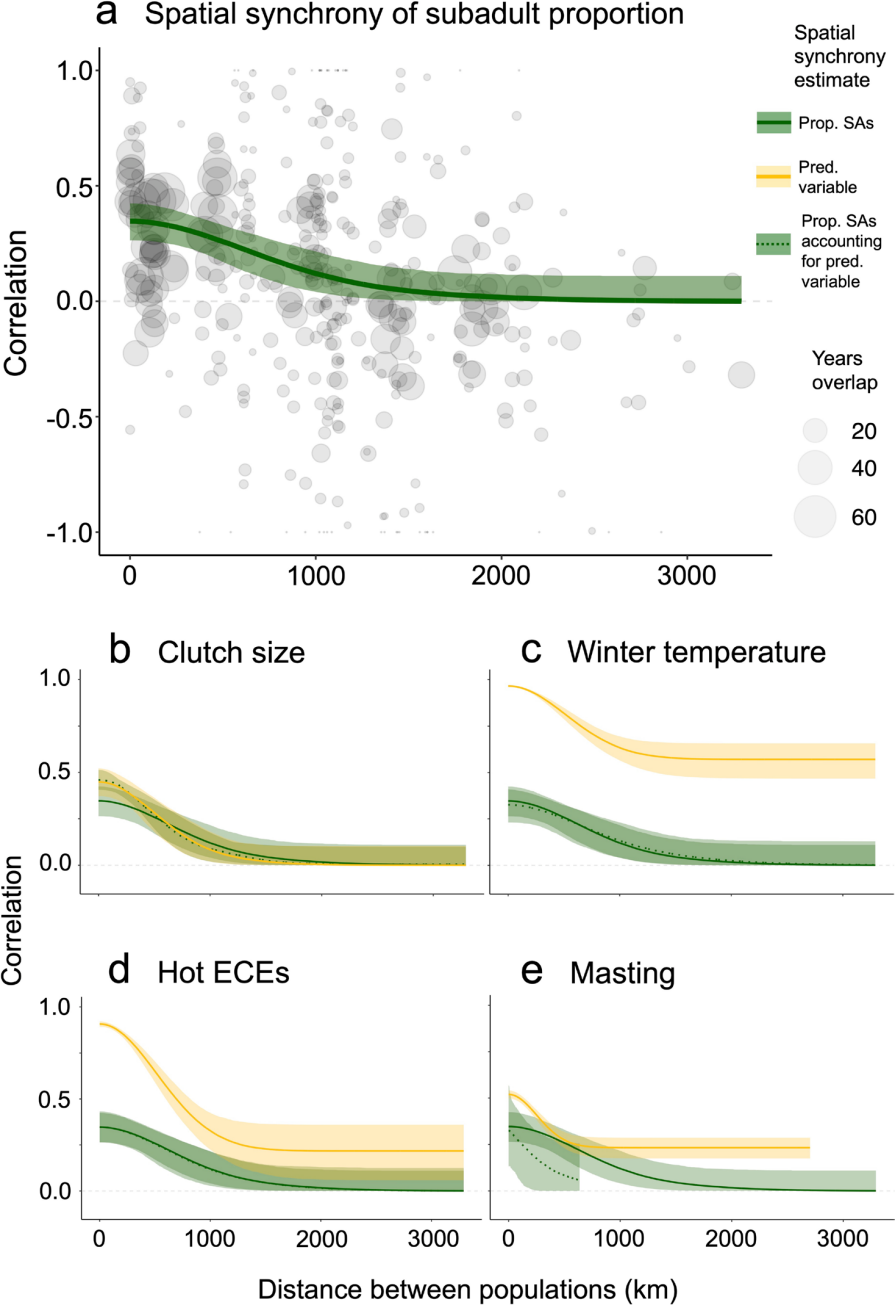
Spatial synchrony of temporal variation in breeding demographic structure in relation to distance between great tit populations. In all plots, distance between populations (km) is on the x‐axis and correlation between paired sites is on the y‐axis. (a) Shows spatial synchrony of temporal fluctuations in the proportion of subadults, where the green line is the median estimate of spatial synchrony (calculated in Equation (2)) based on 2000 bootstrap replicates, with light green shading representing 95% credible intervals, and point size relative to the number of years of overlap between time series of pairwise sites. In (b–e), the dark green solid line is the estimate of spatial synchrony in the proportion of subadults with 95% credible intervals, the yellow line is the spatial synchrony of the given predictor (reproductive or environmental) variable, and the green dashed line is the spatial synchrony in the proportion of subadults once accounting for the given reproductive or environmental variable.

We found large‐scale synchrony in the proportion of breeding subadults, which decreased as distance between populations increased (${\hat{\rho }}_{100\mathrm{km}}$ = 0.340 [0.260, 0.416]; ${\hat{\rho }}_{500\mathrm{km}}$ = 0.253 [0.163, 0.330]; ${\hat{\rho }}_{2500\mathrm{km}}$ = 0.004 [< 0.001, 0.112]; Figure 3a; Table 1), and a large estimate for the scale of spatial autocorrelation ($\hat{l}$ = 641 km [371 km, 1000 km]). Spatial synchrony was very similar for all alternative descriptors of demographic structure (Supporting Information S1; Table S3; Figure S7). Additionally, spatial synchrony was similar in recent years compared to over all time (Table S5; Figure S9) and the general pattern of synchrony appeared similar when using a semi‐parametric approach which allowed for negative synchrony on average (Table S6; Figure S10).

**TABLE 1:** Spatial synchrony of temporal variation in breeding demographic structure across great tit populations.

Parameter	Median	95% credible intervals
$\rho_{0}$	0.344	[0.264, 0.424]
$\rho_{\infty }$	< 0.001	[< 0.001, 0.112]
l	641 km	[371 km, 1000 km]
$\rho_{100\mathrm{km}}$	0.340	[0.260, 0.416]
$\rho_{500\mathrm{km}}$	0.253	[0.163, 0.330]
$\rho_{1000\mathrm{km}}$	0.115	[0.035, 0.205]
$\rho_{2500\mathrm{km}}$	0.004	[< 0.001, 0.112]

*Note:* Estimates are provided for spatial synchrony parameters (calculated in Equation (2)); and for synchrony at distances of 100 km, 500 km, 1000 km and 2500 km.

Interestingly, neither clutch size nor many of the environmental variables explained spatial synchrony in breeding demographic structure (Figure 3b–d; Table S7). There is some evidence that masting had a synchronising effect on the subadult proportion for populations within 100 km of mast data collection (${\hat{\rho }}_{100\mathrm{km}}$ = 0.239 [0.030, 0.419]; ${\hat{\rho }}_{500\mathrm{km}}$ = 0.109 [< 0.001, 0.303]; Figure 3e). However, these results should be interpreted with caution given that the synchrony estimates are based on a subset of populations across which the maximum distance between populations that overlap their time series (635 km) is similar to the estimated spatial scale of synchrony in demographic structure across all populations.

## 4 Discussion

Using 77,964 individuals across 32 great tit populations collectively monitored over 702 years, we show that reproductive and environmental variables covary with breeding demographic structure, with average clutch size from previous breeding, winter temperature, and variation in beech masting being the strongest predictors of demographic structure. We report distance‐dependent synchrony in breeding demographic structure, which is maintained at approximately 650 km. However, despite association between reproductive and environmental factors with populations' demographic structure, we did not find support for these factors in explaining synchrony (except for some evidence that beech masting partially synchronises fluctuations).

### 4.1 Temporal Variation in Breeding Demographic Structure and Explanatory Variables

Variation in age structure can have important consequences for demographic and social functioning (Coulson, Gaillard, and Festa‐Bianchet 2005; Gamelon et al. 2019; Siracusa et al. 2023; Woodman et al. 2024), yet little research has linked its variation across multiple populations directly to reproductive and environmental variability. This is because much research has focussed on how variation in population vital rates covary with environmental factors in single populations (Coulson, Milner‐Gulland, and Clutton‐Brock 2000; Coulson et al. 2001; Farand, Allainé, and Coulon 2002), without explicitly linking this to variation in demographic structure (Hoy et al. 2020). Here, we provide evidence that reproductive and environmental variability influences breeding population demographic structure through affecting the proportion of subadults found breeding annually.

The strongest predictor of demographic structure was average clutch size, which predicts younger breeding populations the following year when clutch sizes are larger, suggesting that on average larger clutch sizes lead to more recruits (Ahola et al. 2009). Additionally, greater fecundity in great tits is linked to higher mortality (Payevsky 2006; Sæther 1988), thus there may be a relative increase in the proportion of subadults the following year if there is high mortality among older individuals due to the cost of producing larger clutches. Directly linking variation in average clutch size and breeding demographic structure reveals an important aspect of fluctuating dynamics in great tit populations. This is because larger clutch sizes are produced when populations are smaller (Kluijver 1951; Lack 1952). Conversely, following an increase in the proportion of subadults due to larger clutch sizes (as shown here), density‐dependence will be strengthened, not only due increased population size, but also because subadults have the strongest effect on density‐dependent regulation, reducing recruitment and survival (Gamelon et al. 2016; Tinbergen, van Balen, and van Eck 1985) and producing fewer fledglings (Perrins and McCleery 1985).

We report a relationship between breeding demographic structure and temperature, but not precipitation, where warmer summers and winters, and more frequent hot ECEs, are associated with smaller proportions of subadults. This is contrary to what might be expected where harsher winters would lead to elevated mortality of inexperienced subadults. Multiple hypotheses might explain why lower temperatures result in a differential response of demographic structure compared to this expectation. For example, it has been shown that the reduction of fat following cold temperatures is not age‐specific in great tits (Gosler 2002). Thus, colder winters might potentially lead to higher mortality rates in older individuals with lower basal metabolic rates (Broggi et al. 2010) or senescence in other physiological traits (Bouwhuis et al. 2012). However, a more plausible hypothesis is that cold temperature‐driven mortality is age‐independent, thus reducing local population size across all ages (van Balen 1980; Kluijver 1951; Payevsky 2006). In high‐quality great tit habitats (as in many populations assessed here), there are often more individuals than available territories where more dominant individuals acquire the breeding sites (Perrins 1979). Great tits resident to a site are more dominant (Krebs 1982; Sandell and Smith 1991), thus upon their death, this likely makes more territories available to subdominant individuals dispersing from surrounding lower‐quality sites (Verhulst, Perrins, and Riddington 1997). This might therefore increase the number of breeding immigrants following high rates of local mortality, for example, due to harsh winters (Grøtan et al. 2009; Tufto et al. 2005). Dispersal between birth and first breeding (natal dispersal) covers greater distances than dispersal between breeding attempts (Greenwood and Harvey 1982). Thus, great tits moving into new environments are often subadults (Greenwood 1980). This therefore might generate an indirect relationship between colder winters and larger proportions of breeding subadults the following spring. Further work should test this hypothesis by assessing winter temperature conditions under which immigration increases in great tits and other non‐migratory species with sink populations in patchy environments.

We found larger beech crops are followed by breeding seasons with larger proportions of subadults. Beech mast is important for winter survival in great tits, and previous work shows it elevates survival particularly in the first‐year of life (Clobert et al. 1988; Källander 1981; Perdeck, Visser, and van Balen 2000). However, such studies assess either single populations, or populations close to one another, thus there is limited understanding of how important masting is for tit demography on a continental‐scale (but see Sæther et al. (2007) for its influence on size of populations located several hundred kilometres apart). Moreover, when we restricted analyses to populations within 100 km of beech data collection, we found a greater effect of masting on breeding demographic structure. This could be due to two reasons: either data collected far from focal populations did not represent actual masting conditions experienced (although there is high spatial synchrony in beech crop cycles, Bogdziewicz et al. 2021); or some populations are in habitats with no or a lower density of beech, thus masting cannot influence demographic fluctuations. Indeed, when assessing population‐specific trends, we find marked variation in the effect of masting on demographic structure (Figure S10 *l*). Further, the strength of this relationship covaries with longitude, which approximates to the European distribution of beech (Figure S1; S12; S13). Although it has been suggested that masting‐related tit population dynamics might be underpinned by synchronous fruiting of multiple tree species that elevate survival across wide‐ranging habitats (Klomp 1980; Perrins 1966), our results may indicate that it is specifically beech which links variation in fruiting cycles with subadult survival.

### 4.2 Spatial Synchrony of Variation in Breeding Demographic Structure

Formally comparing our estimates of synchrony with population characteristics from previous studies, we find that the scale of synchrony in breeding demographic structure ($\hat{l}$ = 641 km) is greater than that of population size (34 km, Sæther et al. 2007) and number of fledglings produced per pair (141 km, Vriend et al. 2023) across the same species. Given the interrelated dynamics between reproductive output, demographic structure, and population abundance (Gamelon et al. 2016, 2019), the differences found in the scale of synchrony across these attributes are interesting and warrant further research. Specifically, incorporating population‐specific density‐dependent models into analysis and quantifying spatial synchrony thereafter might shed light as to how these different population characteristics interact to affect the scale of spatial synchrony in each other, and influence the synchronising effect of environmental variability on population dynamics (see later discussion).

Despite covariation between reproductive and environmental factors with breeding demographic structure (Figure 2), accounting for such variables did not significantly affect the scale of synchrony, other than evidence that beech masting may contribute to synchronising fluctuations in the subadult proportion (Figure 3b–e; Table S7). For any collection of populations, it is not necessarily expected that the same environmental variables would uniformly influence their demographic structures between years or synchronise fluctuations over time. An extensive body of demographic theory demonstrates that density‐dependence can lead to complex dynamics, including chaos, in discrete population structures (Caswell 2000; Hastings et al. 1993; Levin 1981). Implicit in our analysis is the assumption that the populations have a fixed‐point (stable) equilibrium demographic structure to which they gravitate, and that all populations are reasonably close to their equilibria. Even if the populations' structures do have a stable equilibrium, they are likely at different distances from their equilibria at any point in time, and thus going through different phases of transient dynamics (Hastings et al. 2018; Koons et al. 2005). If so, environmental effects may be obscured by internal demographic processes that might be on divergent or uncorrelated trajectories. While these points serve as caveats concerning the complex dynamics influencing the populations, they also underscore the pronounced impact exerted by the explanatory variables (e.g., beech masting) on breeding demographic structure, penetrating through the complicating dynamics.

Specifically, populations within 100 km of beech data collection had lower estimates of synchrony in breeding demographic structure once variation in masting had been accounted for. This may indicate that synchrony in beech crop cycles (Bogdziewicz et al. 2021) act to synchronise fluctuations in the proportion of breeding populations consisting of subadults, but only in populations that breed within the distribution of beech. Further, this might suggest an indirect effect of spatial autocorrelation in weather in synchronising demographic structure, seeing as temperature and precipitation synchronise beech crop cycles (Bogdziewicz et al. 2021). Given the effects of climate warming on beech, which has increased overall seed production but reduced reproductive synchrony among individuals (Bogdziewicz et al. 2020), we might expect populations which are seed predators of beech, such as great tits, to have reduced synchrony in demographic structure with increasing climate warming. This is particularly pertinent given that masting affects population dynamics across many taxa (Bogdziewicz, Zwolak, and Crone 2016).

There was a general lack of an effect from other environmental variables in synchronising breeding demographic structure. Spatial synchrony is generated either through dispersal between populations; interspecific trophic interactions with other spatially‐synchronised populations; or a common influence from spatially autocorrelated environmental variables (the Moran effect). Dispersal between the assessed populations is unlikely to play an important role here, as great tits disperse over smaller spatial scales compared to our estimate of the scale of synchrony (Greenwood, Harvey, and Perrins 1979; Tufto et al. 2005). However, dispersal more broadly might synchronise demographic structure fluctuations if there are simultaneous annual irruptive waves of subadult immigrants that move into the assessed sites prior to breeding (Nowakowski and Vähätalo 2003), especially as annual variation in such waves correlate with years of high recruitment (Grøtan et al. 2009).

Our results provide some evidence that the Moran effect may not significantly underpin synchrony in breeding demographic structure with regard to the climatic variables assessed here, because individually accounting for such variables does not substantially reduce estimates of synchrony. This may suggest that while local climatic variability in the focal variables might drive demographic shifts within populations, their broad‐scale spatial autocorrelation may not synchronise variation in demographic structure across populations. Thus, we might not expect wide‐scale changes to these variables induced by climate change to synchronously affect demographic structure in this species. However, it is worth noting that our approach evaluates the role of climatic variables in isolation. As such, while the role of single climatic variables in synchronising demographic structure seems to be absent, multiple climatic factors may interact to induce synchrony. Investigating the combined effect of multiple climatic variables on spatial synchrony would be a valuable avenue for future research as methods evolve to include this complexity within a similar framework presented here.

Extending our approach to species with alternative life‐history and ecological strategies might also further our understanding on the role of climate in synchronising age structure. For example, here we study resident annual breeders with few discrete age‐cohorts. It might be expected that in even shorter‐lived species with fewer age‐cohorts and more irruptive population dynamics (such as where recruitment is dependent on age‐specific dispersal over highly variable environments), we might expect a greater synchronising effect of single climatic variables on demographic structure between populations, possibly leading to a greater effect of climate change on population dynamics across populations.

Given our findings, other non‐climatic factors may play a role in synchronising great tit breeding demographic structure. The Moran effect traditionally emphasises abiotic climatic drivers of synchrony, yet biotic factors, such as predator or prey population dynamics, might synchronise demographic structure while themselves being influenced by spatially autocorrelated climatic conditions. For example, tits are highly susceptible to predation in the first month of fledging (Naef‐Daenzer, Widmer, and Nuber 2001; Perrins and Geer 1980). Thus, if there is spatial synchrony in predator population dynamics, this might induce synchrony in breeding demographic structure through its effects on survival of subadults prior to breeding during the first year of life. Additionally, post‐fledging food availability is important for survival (Drent 1984; Payevsky 2006), which might influence the proportion of subadults found breeding the following season. Diet during this period consists predominantly of caterpillars (Verhulst and Hut 1996), the availability of which will not only be influenced by weather, but other factors that affect invertebrate abundance and phenology, such as habitat heterogeneity and density‐dependence. Exploring spatial synchrony in demographic structure while considering multiple trophic levels could provide a deeper understanding of how biotic and abiotic factors collectively shape demographic structure synchrony.

Ecological features might not just act to synchronise breeding demographic structure through effects on age‐specific survival, but also through effects on reproduction. For example, great tit reproductive rates vary along an urban–non‐urban gradient (Bukor et al. 2022; Charmantier et al. 2017; Corsini et al. 2021), and reproductive responses to weather depend on whether breeding takes place in urban or non‐urban habitats (Saulnier et al. 2023). Thus, if populations that are closer experience more similar habitats, this might induce spatial synchrony in their population dynamics.

Finally, we should expect that fluctuations in demographic structure are not fully explained by variation in environmental variables because of population‐specific density‐dependent dynamics. Local density‐dependence can affect reproductive responses to environmental stochasticity (Møller et al. 2020), and different dynamics reduces spatial synchrony between populations (Hugueny 2006; Walter et al. 2017). Thus, the spatial scale at which density‐dependence remains similar between populations should influence the interaction between demographic structure and environmental variability, and the resultant spatial synchrony of fluctuations in breeding demographic structure.

## 5 Conclusions

Using multiple overlapping time series, our study quantifies associations between reproductive and environmental variables with breeding demographic structure in 32 spatially‐distinct wild great tit populations. We report spatial synchrony of fluctuations in breeding demographic structure at approximately 650 km, but find little evidence that accounting for variation in environmental factors reduces the scale of synchrony, apart from a synchronising effect of beech masting. Further research should focus on how additional ecological features and population‐specific density‐dependent dynamics may contribute to the observed spatial scale of synchrony in demographic structure found here. Moreover, considering different species with varying life‐history and ecological strategies could provide broader insights into the mechanisms driving synchrony in natural populations.

## Author Contributions

J.P.W., J.A.F. and B.C.S. conceived the study. J.P.W. analysed the data, with significant contributions from S.J.G.V. J.P.W. wrote the first draft of the manuscript, with substantial input from J.A.F. and B.C.S. All other authors collected and provided data and/or comments on later drafts of the manuscript.

### Peer Review

The peer review history for this article is available at https://www.webofscience.com/api/gateway/wos/peer‐review/10.1111/ele.70079.

## Supporting information


Data S1.

## Data Availability

Data and code archived in a Dryad digital repository (https://doi.org/10.5061/dryad.k0p2ngfgg).

## References

[ele70079-bib-0001] Abbott, K. C. 2007. “Does the Pattern of Population Synchrony Through Space Reveal if the Moran Effect Is Acting?” Oikos 116: 903–912.

[ele70079-bib-0002] Ahola, M. P. , T. Laaksonen , T. Eeva , and E. Lehikoinen . 2009. “Great Tits Lay Increasingly Smaller Clutches Than Selected for: A Study of Climate‐ and Density‐Related Changes in Reproductive Traits.” Journal of Animal Ecology 78: 1298–1306. 19682140 10.1111/j.1365-2656.2009.01596.x

[ele70079-bib-0003] Bjørnstad, O. N. , R. A. Ims , and X. Lambin . 1999. “Spatial Population Dynamics: Analyzing Patterns and Processes of Population Synchrony.” Trends in Ecology & Evolution 14: 427–432. 10511718 10.1016/s0169-5347(99)01677-8

[ele70079-bib-0004] Bjørnstad, O. N. , N. C. Stenseth , and T. Saitoh . 1999. “Synchrony and Scaling in Dynamics of Voles and Mice in Northern Japan.” Ecology 80: 622–637.

[ele70079-bib-0005] Bogdziewicz, M. , A. Hacket‐Pain , D. Ascoli , and J. Szymkowiak . 2021. “Environmental Variation Drives Continental‐Scale Synchrony of European Beech Reproduction.” Ecology 102: 1–10. 10.1002/ecy.3384 33950521

[ele70079-bib-0006] Bogdziewicz, M. , D. Kelly , P. A. Thomas , J. G. A. Lageard , and A. Hacket‐Pain . 2020. “Climate Warming Disrupts Mast Seeding and Its Fitness Benefits in European Beech.” Nature Plants 6: 88–94. 32042155 10.1038/s41477-020-0592-8

[ele70079-bib-0007] Bogdziewicz, M. , R. Zwolak , and E. E. Crone . 2016. “How Do Vertebrates Respond to Mast Seeding?” Oikos 125: 300–307.

[ele70079-bib-0008] Bolte, A. , T. Czajkowski , and T. Kompa . 2007. “The North‐Eastern Distribution Range of European Beech ‐ a Review.” Forestry 80: 413–429.

[ele70079-bib-0010] Bouwhuis, S. , R. Choquet , B. C. Sheldon , and S. Verhulst . 2012. “The Forms and Fitness Cost of Senescence: Age‐Specific Recapture, Survival, Reproduction, and Reproductive Value in a Wild Bird Population.” American Naturalist 179: E15–E27. 10.1086/663194 22173469

[ele70079-bib-0011] Bouwhuis, S. , B. C. Sheldon , S. Verhulst , and A. Charmantier . 2009. “Great Tits Growing Old: Selective Disappearance and the Partitioning of Senescence to Stages Within the Breeding Cycle.” Proceedings of the Royal Society B: Biological Sciences 276: 2769–2777. 10.1098/rspb.2009.0457 PMC2839957 19403537

[ele70079-bib-0012] Broggi, J. , E. Hohtola , K. Koivula , M. Orell , and J. Å. Nilsson . 2010. “Idle Slow as You Grow Old: Longitudinal Age‐Related Metabolic Decline in a Wild Passerine.” Evolutionary Ecology 24: 177–184.

[ele70079-bib-0013] Bukor, B. , G. Seress , I. Pipoly , et al. 2022. “Double‐Brooding and Annual Breeding Success of Great Tits in Urban and Forest Habitats.” Current Zoology 68: 517–525. 36324531 10.1093/cz/zoab096 PMC9616069

[ele70079-bib-0014] Bürkner, P.‐C. 2017. “Brms: An R Package for Bayesian Multilevel Models Using Stan.” Journal of Statistical Software 80: 1–28.

[ele70079-bib-0015] Caswell, H. 2000. Matrix Population Models: Construction, Analysis, and Interpretation. 2nd ed. Sinauer Associates.

[ele70079-bib-0016] Charmantier, A. , V. Demeyrier , M. Lambrechts , S. Perret , and A. Grégoire . 2017. “Urbanization Is Associated With Divergence in Pace‐Of‐Life in Great Tits.” Frontiers in Ecology and Evolution 5: 53. 10.3389/fevo.2017.00053.

[ele70079-bib-0017] Clobert, J. , C. M. Perrins , R. H. McCleery , and A. G. Gosler . 1988. “Survival Rate in the Great Tit *Parus major* in Relation to Sex, Age, and Immigration Status.” Journal of Animal Ecology 57: 287–306.

[ele70079-bib-0018] Cornes, R. C. , G. van der Schrier , E. J. M. van den Besselaar , and P. D. Jones . 2018. “An Ensemble Version of the E‐OBS Temperature and Precipitation Data Sets.” Journal of Geophysical Research: Atmospheres 123: 9391–9409.

[ele70079-bib-0019] Corsini, M. , E. M. Schöll , I. Di Lecce , M. Chatelain , A. Dubiec , and M. Szulkin . 2021. “Growing in the City: Urban Evolutionary Ecology of Avian Growth Rates.” Evolutionary Applications 14: 69–84. 33519957 10.1111/eva.13081 PMC7819560

[ele70079-bib-0020] Coulson, T. , E. A. Catchpole , S. D. Albon , et al. 2001. “Age, Sex, Density, Winter Weather, and Population Crashes in Soay Sheep.” Science 292: 1528–1531. 11375487 10.1126/science.292.5521.1528

[ele70079-bib-0021] Coulson, T. , J. M. Gaillard , and M. Festa‐Bianchet . 2005. “Decomposing the Variation in Population Growth Into Contributions From Multiple Demographic Rates.” Journal of Animal Ecology 74: 789–801.

[ele70079-bib-0022] Coulson, T. , F. Guinness , J. Pemberton , and T. H. Clutton‐Brock . 2004. “The Demographic Consequences of Releasing a Population of Red Deer From Culling.” Ecology 85: 411–422.

[ele70079-bib-0023] Coulson, T. , E. J. Milner‐Gulland , and T. Clutton‐Brock . 2000. “The Relative Roles of Density and Climatic Variation on Population Dynamics and Fecundity Rates in Three Contrasting Ungulate Species.” Proceedings of the Royal Society B: Biological Sciences 267: 1771–1779. 10.1098/rspb.2000.1209 PMC1690729 12233776

[ele70079-bib-0024] Culina, A. , F. Adriaensen , L. D. Bailey , et al. 2021. “Connecting the Data Landscape of Long‐Term Ecological Studies: The SPI‐Birds Data Hub.” Journal of Animal Ecology 90: 2147–2160. 33205462 10.1111/1365-2656.13388 PMC8518542

[ele70079-bib-0025] Drent, P. J. 1984. “Mortality and Dispersal in Summer and Its Consequences for the Density of Great Tits *Parus major* at the Onset of Autumn.” Ardea 72: 127–162.

[ele70079-bib-0026] Elton, C. S. 1924. “Periodic Fluctuations in the Numbers of Animals: Their Causes and Effects.” British Journal of Experimental Biology 2: 119–163.

[ele70079-bib-0027] Engen, S. , R. Lande , and B.‐E. Sæther . 2002. “The Spatial Scale of Population Fluctuations and Quasi‐Extinction Risk.” American Naturalist 160: 439–451. 10.1086/342072 18707521

[ele70079-bib-0028] Engen, S. , R. Lande , B.‐E. Seæther , and T. Bregnballe . 2005. “Estimating the Pattern of Synchrony in Fluctuating Populations.” Journal of Animal Ecology 74: 601–611.

[ele70079-bib-0029] Farand, É. , D. Allainé , and J. Coulon . 2002. “Variation in Survival Rates for the Alpine Marmot (*Marmota Marmota*): Effects of Sex, Age, Year, and Climatic Factors.” Canadian Journal of Zoology 80: 342–349.

[ele70079-bib-0030] Gamelon, M. , V. Grøtan , S. Engen , E. Bjørkvoll , M. E. Visser , and B. E. Sæther . 2016. “Density Dependence in an Age‐Structured Population of Great Tits: Identifying the Critical Age Classes.” Ecology 97: 2479–2490. 27859080 10.1002/ecy.1442

[ele70079-bib-0031] Gamelon, M. , S. J. G. Vriend , S. Engen , et al. 2019. “Accounting for Interspecific Competition and Age Structure in Demographic Analyses of Density Dependence Improves Predictions of Fluctuations in Population Size.” Ecology Letters 22: 797–806. 30816630 10.1111/ele.13237

[ele70079-bib-0032] Genz, A. , F. Bretz , T. Miwa , et al. 2021. “Package ‘mvtnorm’.” Journal of Computational and Graphical Statistics 11: 155.

[ele70079-bib-0033] Gosler, A. G. 2002. “Strategy and Constraint in the Winter Fattening Response to Temperature in the Great Tit *Parus major* .” Journal of Animal Ecology 71: 771–779.

[ele70079-bib-0034] Gosler, A. 1993. The Great Tit. Hamlyn, London.

[ele70079-bib-0035] Greenwood, P. J. 1980. “Mating Systems, Philopatry and Dispersal in Birds and Mammals.” Animal Behaviour 28: 1140–1162.

[ele70079-bib-0036] Greenwood, P. J. , and P. H. Harvey . 1982. “The Natal and Breeding Dispersal of Birds.” Annual Review of Ecology and Systematics 13: 1–21.

[ele70079-bib-0037] Greenwood, P. J. , P. H. Harvey , and C. M. Perrins . 1979. “The Role of Dispersal in the Great Tit (*Parus major*): The Causes, Consequences and Heritability of Natal Dispersal.” Journal of Animal Ecology 48: 123–142.

[ele70079-bib-0038] Grøtan, V. , B. Sæther , S. Engen , J. H. van Balen , A. C. Perdeck , and M. E. Visser . 2009. “Spatial and Temporal Variation in the Relative Contribution of Density Dependence, Climate Variation and Migration to Fluctuations in the Size of Great Tit Populations.” Journal of Animal Ecology 78: 447–459. 19302127 10.1111/j.1365-2656.2008.01488.x

[ele70079-bib-0039] Grøtan, V. , B.‐E. Sæther , S. Engen , et al. 2005. “Climate Causes Large‐Scale Spatial Synchrony in Population Fluctuations of a Temperate Herbivore.” Ecology 86: 1472–1482.

[ele70079-bib-0040] Hacket‐Pain, A. , J. J. Foest , I. S. Pearse , et al. 2022. “MASTREE+: Time‐Series of Plant Reproductive Effort From Six Continents.” Global Change Biology 28: 3066–3082. 35170154 10.1111/gcb.16130 PMC9314730

[ele70079-bib-0041] Hansen, B. B. , V. Grøtan , I. Herfindal , and A. M. Lee . 2020. “The Moran Effect Revisited: Spatial Population Synchrony Under Global Warming.” Ecography (Copenhagen, Denmark) 43: 1591–1602.

[ele70079-bib-0042] Harvey, P. H. , P. J. Greenwood , C. M. Perrins , and A. R. Martin . 1979. “Breeding Success of Great Tits *Parus major* in Relation to Age of Male and Female Parent.” Ibis (London, England) 121: 216–219.

[ele70079-bib-0043] Hastings, A. , K. C. Abbott , K. Cuddington , et al. 2018. “Transient Phenomena in Ecology.” Science 361: 6412. 10.1126/science.aat6412 30190378

[ele70079-bib-0044] Hastings, A. , C. L. Hom , S. Ellner , P. Turchin , and H. C. J. Godfray . 1993. “Chaos in Ecology: Is Mother Nature a Strange Attractor?” Annual Review of Ecology and Systematics 24: 1–33.

[ele70079-bib-0045] Heino, M. , V. Kaitala , E. Ranta , and J. Lindstrom . 1997. “Synchronous Dynamics and Rates of Extinction in Spatially Structured Populations.” Proceedings of the Royal Society B: Biological Sciences 264: 481–486.

[ele70079-bib-0046] Herfindal, I. , T. Tveraa , A. Stien , E. J. Solberg , and V. Grøtan . 2020. “When Does Weather Synchronize Life‐History Traits? Spatiotemporal Patterns in Juvenile Body Mass of Two Ungulates.” Journal of Animal Ecology 89: 1419–1432. 32108334 10.1111/1365-2656.13192

[ele70079-bib-0047] Hoy, S. R. , D. R. MacNulty , D. W. Smith , et al. 2020. “Fluctuations in Age Structure and Their Variable Influence on Population Growth.” Functional Ecology 34: 203–216.

[ele70079-bib-0050] Hugueny, B. 2006. “Spatial Synchrony in Population Fluctuations: Extending the Moran Theorem to Cope With Spatially Heterogeneous Dynamics.” Oikos 115: 3–14.

[ele70079-bib-0051] Ims, R. A. , and H. P. Andreassen . 2000. “Spatial Synchronization of Vole Population Dynamics by Predatory Birds.” Nature 408: 194–196. 11089971 10.1038/35041562

[ele70079-bib-0052] Jarillo, J. , B. E. Sæther , S. Engen , and F. J. Cao . 2018. “Spatial Scales of Population Synchrony of Two Competing Species: Effects of Harvesting and Strength of Competition.” Oikos 127: 1459–1470.

[ele70079-bib-0053] Jones, J. , P. J. Doran , and R. T. Holmes . 2003. “Climate and Food Synchronize Regional Forest Bird Abundances.” Ecology 84: 3024–3032.

[ele70079-bib-0054] Källander, H. 1981. “The Effects of Provision of Food in Winter on a Population of the Great Tit *Parus major* and the Blue tit *P. Caeruleus* .” Ornis Scandinavica 12: 244–248.

[ele70079-bib-0055] Kelly, D. 1994. “The Evolutionary Ecology of Mast Seeding.” Trends in Ecology & Evolution 9: 465–470. 21236924 10.1016/0169-5347(94)90310-7

[ele70079-bib-0056] Kendall, B. E. , O. N. Bjørnstad , J. Bascompte , T. H. Keitt , and W. F. Fagan . 2000. “Dispersal, Environmental Correlation, and Spatial Synchrony in Population Dynamics.” American Naturalist 155: 628–636. 10.1086/303350 10777435

[ele70079-bib-0057] Klomp, H. 1980. “Fluctuations and Stability in Great Tit Populations.” Ardea. Revista del Club Español de Ornitología 68: 205–224.

[ele70079-bib-0058] Kluijver, H. N. 1951. “The Population Ecology of the Great Tit, Parus m. Major L.” Ardea 39: 1–135.

[ele70079-bib-0059] Koenig, W. D. 1999. “Spatial Autocorrelation of Ecological Phenomena.” Trends in Ecology & Evolution 14: 22–26. 10234243 10.1016/s0169-5347(98)01533-x

[ele70079-bib-0116] Koenig, W. D ., and A. M. Liebhold . 2016. “Temporally Increasing Spatial Synchrony of North American Temperature and Bird Populations.” Nature Climate Change 6: 614–617.

[ele70079-bib-0060] Koons, D. N. , J. B. Grand , B. Zinner , and R. F. Rockwell . 2005. “Transient Population Dynamics: Relations to Life History and Initial Population State.” Ecological Modelling 185: 283–297.

[ele70079-bib-0061] Koons, D. N. , D. T. Iles , M. Schaub , and H. Caswell . 2016. “A Life‐History Perspective on the Demographic Drivers of Structured Population Dynamics in Changing Environments.” Ecology Letters 19: 1023–1031. 27401966 10.1111/ele.12628

[ele70079-bib-0062] Krebs, J. R. 1982. “Territorial Defence in the Great Tit (*Parus major*): Do Residents Always Win?” Behavioral Ecology and Sociobiology 11: 185–194.

[ele70079-bib-0063] Lack, D. 1952. “Reproductive Rate and Population Density in the Great Tit: Kluijvers Study.” Ibis (London, England) 94: 167–173.

[ele70079-bib-0064] Lande, R. , S. Engen , and B.‐E. Sæther . 1999. “Spatial Scale of Population Synchrony: Environmental Correlation Versus Dispersal and Density Regulation.” American Naturalist 154: 271–281. 10.1086/303240 10506543

[ele70079-bib-0065] Levin, S. A. 1981. “Age‐Structure and Stability in Multiple‐Age Spawning Populations.” In Renewable Resource Management: Proceedings of a Workshop on Control Theory Applied to Renewable Resource Management and Ecology Held in Christchurch, New Zealand January 7–11, 1980, 21–45. Springer.

[ele70079-bib-0066] Liebhold, A. , W. D. Koenig , and O. N. Bjørnstad . 2004. “Spatial Synchrony in Population Dynamics.” Annual Review of Ecology, Evolution, and Systematics 35: 467–490.

[ele70079-bib-0067] Lillegård, M. , S. Engen , and B.‐E. Sæther . 2005. “Bootstrap Methods for Estimating Spatial Synchrony of Fluctuating Populations.” Oikos 109: 342–350.

[ele70079-bib-0068] Marrot, P. , D. Garant , and A. Charmantier . 2017. “Multiple Extreme Climatic Events Strengthen Selection for Earlier Breeding in a Wild Passerine.” Philosophical Transactions of the Royal Society, B: Biological Sciences 372: 1–9. 10.1098/rstb.2016.0372 PMC5434099 28483864

[ele70079-bib-0069] Mills, L. S. 2012. Conservation of Wildlife Populations: Demography, Genetics, and Management. John Wiley & Sons.

[ele70079-bib-0070] Møller, A. P. , J. Balbontín , A. A. Dhondt , et al. 2020. “Interaction of Climate Change With Effects of Conspecific and Heterospecific Density on Reproduction.” Oikos 129: 1807–1819.

[ele70079-bib-0071] Moran, P. A. P. 1953. “The Statistical Analysis of the Canadian Lynx Cycle. II. Synchronization and Metereology.” Australian Journal of Zoology 1: 291–298.

[ele70079-bib-0072] Moreno, J. , and A. P. Møller . 2011. “Extreme Climatic Events in Relation to Global Change and Their Impact on Life Histories.” Current Zoology 57: 375–389.

[ele70079-bib-0073] Mortelliti, A. , M. Westgate , J. Stein , J. Wood , and D. B. Lindenmayer . 2015. “Ecological and Spatial Drivers of Population Synchrony in Bird Assemblages.” Basic and Applied Ecology 16: 269–278.

[ele70079-bib-0074] Naef‐Daenzer, B. , F. Widmer , and M. Nuber . 2001. “Differential Post‐Fledging Survival of Great and Coal Tits in Relation to Their Condition and Fledging Date.” Journal of Animal Ecology 70: 730–738.

[ele70079-bib-0075] Nowakowski, J. K. , and A. V. Vähätalo . 2003. “Is the Great Tit *Parus major* an Irruptive Migrant in North‐Eastern Europe?” Ardea 91: 231–243.

[ele70079-bib-0076] Olin, A. B. , N. S. Banas , P. J. Wright , M. R. Heath , and R. G. Nager . 2020. “Spatial Synchrony of Breeding Success in the Black‐Legged Kittiwake *Rissa Tridactyla* Reflects the Spatial Dynamics of Its Sandeel Prey.” Marine Ecology Progress Series 638: 177–190.

[ele70079-bib-0077] Olmos, M. , M. R. Payne , M. Nevoux , et al. 2020. “Spatial Synchrony in the Response of a Long Range Migratory Species ( *Salmo salar* ) to Climate Change in the North Atlantic Ocean.” Global Change Biology 26: 1319–1337. 31701595 10.1111/gcb.14913

[ele70079-bib-0078] Paradis, E. 1997. “Metapopulations and Chaos: On the Stabilizing Influence of Dispersal.” Journal of Theoretical Biology 186: 261–266.

[ele70079-bib-0079] Paradis, E. , S. R. Baillie , W. J. Sutherland , and R. D. Gregory . 1999. “Dispersal and Spatial Scale Affect Synchrony in Spatial Population Dynamics.” Ecology Letters 2: 114–120.

[ele70079-bib-0080] Paradis, E. , S. R. Baillie , W. J. Sutherland , and R. D. Gregory . 2000. “Spatial Synchrony in Populations of Birds: Effects of Habitat, Population Trend, and Spatial Scale.” Ecology 81: 2112–2125.

[ele70079-bib-0081] Payevsky, V. A. 2006. “Mortality Rate and Population Density Regulation in the Great Tit, *Parus major* L.: A Review.” Russian Journal of Ecology 37: 180–187.

[ele70079-bib-0082] Perdeck, A. C. , M. E. Visser , and J. H. van Balen . 2000. “Great Tit *Parus major* Survival, and the Beech‐Crop Cycle.” Ardea 88: 99–106.

[ele70079-bib-0084] Perrins, C. M. 1966. “The Effect of Beech Crops on Great Tit Populations and Movements.” British Birds 59: 419–432.

[ele70079-bib-0085] Perrins, C. M. 1979. British Tits. 1st ed. Collins.

[ele70079-bib-0086] Perrins, C. M. , and T. A. Geer . 1980. “The Effect of Sparrowhawks on Tit Populations.” Ardea 68: 133–142.

[ele70079-bib-0087] Perrins, C. M. , and R. H. McCleery . 1985. “The Effect of Age and Pair Bond on the Breeding Success of Great Tits *Parus major* .” Ibis (London, England) 127: 306–315.

[ele70079-bib-0088] R Core Team . 2021. R: A Language and Environment for Statistical Computing. R Foundation for Statistical Computing.

[ele70079-bib-0089] Ranta, E. , V. Kaitala , J. Lindström , and E. Helle . 1997. “The Moran Effect and Synchrony in Population Dynamics.” Oikos 78: 136–142.

[ele70079-bib-0090] Reuman, D. C. , M. C. N. Castorani , K. C. Cavanaugh , L. W. Sheppard , J. A. Walter , and T. W. Bell . 2023. “How Environmental Drivers of Spatial Synchrony Interact.” Ecography 2023: 1–18.

[ele70079-bib-0091] Ripa, J. 2000. “Analysing the Moran Effect and Dispersal: Their Significance and Interaction in Synchronous Population Dynamics.” Oikos 90: 175–187.

[ele70079-bib-0092] Rollinson, C. R. , A. O. Finley , M. R. Alexander , et al. 2021. “Working Across Space and Time: Nonstationarity in Ecological Research and Application.” Frontiers in Ecology and the Environment 19: 66–72.

[ele70079-bib-0093] Ruxton, G. D. 1994. “Low Levels of Immigration Between Chaotic Populations Can Reduce System Extinctions by Inducing Asynchronous Regular Cycles.” Proceedings of the Royal Society B: Biological Sciences 256: 189–193.

[ele70079-bib-0094] Sæther, B.‐E. 1988. “Pattern of Covariation Between Life‐History Traits of European Birds.” Nature 331: 616–617. 3340211 10.1038/331616a0

[ele70079-bib-0095] Sæther, B.‐E. , S. Engen , V. Grøtan , et al. 2007. “The Extended Moran Effect and Large‐Scale Synchronous Fluctuations in the Size of Great Tit and Blue Tit Populations.” Journal of Animal Ecology 76: 315–325. 17302839 10.1111/j.1365-2656.2006.01195.x

[ele70079-bib-0096] Sandell, M. , and H. G. Smith . 1991. “Dominance, Prior Occupancy, and Winter Residency in the Great Tit (*Parus major*).” Behavioral Ecology and Sociobiology 29: 147–152.

[ele70079-bib-0097] Saulnier, A. , J. Bleu , A. Boos , et al. 2023. “Reproductive Differences Between Urban and Forest Birds Across the Years: Importance of Environmental and Weather Parameters.” Urban Ecosystem 26: 395–410.

[ele70079-bib-0098] Selås, V. 1997. “Cyclic Population Fluctuations of Herbivores as an Effect of Cyclic Seed Cropping of Plants: The Mast Depression Hypothesis.” Oikos 80: 257–268.

[ele70079-bib-0099] Sibly, R. M. , and J. Hone . 2002. “Population Growth Rate and Its Determinants: An Overview.” Philosophical Transactions of the Royal Society, B: Biological Sciences 357: 1153–1170. 10.1098/rstb.2002.1117 PMC1693026 12396508

[ele70079-bib-0100] Siracusa, E. R. , A. S. Pereira , J. B. Brask , et al. 2023. “Ageing in a Collective: The Impact of Ageing Individuals on Social Network Structure.” Philosophical Transactions of the Royal Society, B: Biological Sciences 378: 1–14. 10.1098/rstb.2022.0061 PMC9939263 36802789

[ele70079-bib-0101] Sullivan, B. L. , C. L. Wood , M. J. Iliff , R. E. Bonney , D. Fink , and S. Kelling . 2009. “eBird: A Citizen‐Based Bird Observation Network in the Biological Sciences.” Biological Conservation 142: 2282–2292.

[ele70079-bib-0102] Svensson, L. 1992. Identification Guide to European Passerines. 4th ed. Lars Svensson. British Trust for Ornithology.

[ele70079-bib-0103] Tinbergen, J. M. , J. H. van Balen , and H. M. van Eck . 1985. “Density Dependent Survival in an Isolated Great Tit Population: Kluyvers Data Reanalysed.” Ardea 73: 38–48.

[ele70079-bib-0104] Tufto, J. , T.‐H. Ringsby , A. A. Dhondt , F. Adriaensen , and E. Matthysen . 2005. “A Parametric Model for Estimation of Dispersal Patterns Applied to Five Passerine Spatially Structured Populations.” American Naturalist 165: 13–26. 10.1086/426698 15729635

[ele70079-bib-0105] van Balen, J. H. 1980. “Population Fluctuations of the Great Tit and Feeding Conditions in Winter.” Ardea 55: 143–164.

[ele70079-bib-0106] Vehtari, A. , A. Gelman , D. Simpson , B. Carpenter , and P.‐C. Bürkner . 2021. “Rank‐Normalization, Folding, and Localization: An Improved R^ for Assessing Convergence of MCMC (With Discussion).” Bayesian Analysis 16: 667–718.

[ele70079-bib-0107] Verhulst, S. 1998. “Multiple Breeding in the Great Tit, II. The Costs of Rearing a Second Clutch.” Functional Ecology 12: 132–140.

[ele70079-bib-0108] Verhulst, S. , and R. A. Hut . 1996. “Post‐Fledging Care, Multiple Breeding and the Costs of Reproduction in the Great Tit.” Animal Behavior 51: 957–966.

[ele70079-bib-0109] Verhulst, S. , C. M. Perrins , and R. Riddington . 1997. “Natal Dispersal of Great Tits in a Patchy Environment.” Ecology 78: 864–872.

[ele70079-bib-0110] Visser, M. E. , F. Adriaensen , J. H. Van Balen , et al. 2003. “Variable Responses to Large‐Scale Climate Change in European Parus Populations.” Proceedings of the Royal Society B: Biological Sciences 270: 367–372. 10.1098/rspb.2002.2244 PMC1691249 12639315

[ele70079-bib-0111] Vriend, S. J. G. , V. Grøtan , M. Gamelon , et al. 2023. “Temperature Synchronizes Temporal Variation in Laying Dates Across European Hole‐Nesting Passerines.” Ecology 104: e3908. 36314902 10.1002/ecy.3908 PMC10078612

[ele70079-bib-0112] Walter, J. A. , L. W. Sheppard , T. L. Anderson , et al. 2017. “The Geography of Spatial Synchrony.” Ecology Letters 20: 801–814. 28547786 10.1111/ele.12782

[ele70079-bib-0113] Wan, X. , M. Holyoak , C. Yan , et al. 2022. “Broad‐Scale Climate Variation Drives the Dynamics of Animal Populations: A Global Multi‐Taxa Analysis.” Biological Reviews 97: 2174–2194. 35942895 10.1111/brv.12888

[ele70079-bib-0114] Woodman, J. P. , E. F. Cole , J. A. Firth , C. M. Perrins , and B. C. Sheldon . 2022. “Disentangling the Causes of Age‐Assortative Mating in Bird Populations With Contrasting Life‐History Strategies.” Journal of Animal Ecology 92: 13851. 10.1111/1365-2656.13851 36423201

[ele70079-bib-0115] Woodman, J. P. , S. Gokcekus , K. B. Beck , J. P. Green , D. H. Nussey , and J. A. Firth . 2024. “The Ecology of Ageing in Wild Societies: Linking Age Structure and Social Behaviour.” Philosophical Transactions of the Royal Society, B: Biological Sciences 379: 464. 10.1098/rstb.2022.0464 PMC11513650 39463244

