# Supplementary material for: Continent‐Wide Drivers of Spatial Synchrony in Breeding Demographic Structure Across Wild Great Tit Populations
Source: Ecol Lett. 2025 Feb 18;28(2):e70079. doi: 10.1111/ele.70079 (PMC11834383; doi:10.1111/ele.70079)
Supplement: Supplementary file 1 — Data S1. [file ELE-28-0-s001.pdf]

SUPPORTING INFORMATION – Continent-wide drivers of spatial synchrony in breeding demographic structure across wild great tit populations

Joe P. Woodman[1], Stefan J. G. Vriend[2], Frank Adriaensen[3], Elena Álvarez[4], Alexander Artemyev[5], Emilio Barba[4], Malcolm D. Burgess[6], Samuel P. Caro[7], Laure Cauchard[8,9], Anne Charmantier[7], Ella F. Cole[1], Niels Dingemanse[10], Blandine Doligez[7], Tapio Eeva[11], Simon R. Evans[12,1], Arnaud Grégoire[7], Marcel Lambrechts[7], Agu Leivits[13], András Liker[14,15], Erik Matthysen[3], Markku Orell[16], John S. Park[1], Seppo Rytkönen[16], Juan Carlos Senar[17], Gábor Seress[14,15], Marta Szulkin[18], Kees van Oers[2,19], Emma Vatka[20,16], Marcel E. Visser[2], Josh A. Firth[1,21] & Ben C. Sheldon[1]

[1]Edward Grey Institute of Field Ornithology, Department of Biology, University of Oxford, Oxford, United Kingdom

[2]Department of Animal Ecology, Netherlands Institute of Ecology (NIOO-KNAW), Wageningen, The Netherlands

[3]Evolutionary Ecology Group, Department of Biology, University of Antwerp, Antwerp, Belgium

[4]'Cavanilles' Institute of Biodiversity and Evolutionary Biology, University of Valencia, Paterna, Spain

[5]Institute of Biology, Karelian Research Centre, Russian Academy of Sciences, Petrozavodsk, 185610, Russia

[6]Centre for Research in Animal Behaviour, University of Exeter, Exeter, Devon, United Kingdom

[7]Centre d'Ecologie Fonctionnelle et Evolutive, Univ Montpellier, CNRS, EPHE, IRD, Montpellier, France

[8]CNRS, Department of Biometry and Evolutionary Biology (LBBE), University of Lyon, University Lyon 1, Villeurbanne, France

[9]Anthropogenic Effects Research Group, Swiss Ornithological Institute, Sempach, Switzerland

[10]Behavioural Ecology, Department of Biology, Ludwig Maximilians University of Munich, Planegg-Martinsried, Germany

[11]Department of Biology, University of Turku, Finland

[12]Centre for Ecology and Conservation, University of Exeter, Cornwall Campus, Penryn TR10 9FE, United Kingdom

[13]Department of Wildlife, Environmental Board, Pärnu, Estonia

[14]Behavioural Ecology Research Group, Center for Natural Sciences, University of Pannonia, Veszprém, Hungary

[15]HUN-REN-PE Evolutionary Ecology Research Group, University of Pannonia, Veszprém, Hungary

[16]Ecology and Genetics Research Unit, Faculty of Science, University of Oulu, Oulu, Finland

[17]Museu Ciències Naturals, Barcelona, Spain

[18]Institute of Evolutionary Biology, Faculty of Biology, Biological and Chemical Research Centre, University of Warsaw, Warsaw, Poland
[19]Behavioural Ecology Group, Wageningen University & Research (WUR), Wageningen, The Netherlands
[20]Research Programme in Organismal and Evolutionary Biology, Faculty of Biological and Environmental Sciences, University of Helsinki, Helsinki, Finland
[21]School of Biology, University of Leeds, Leeds, United Kingdom

Table of contents

Section	Page
**1. Supporting methods**	**3**
Study systems and data collection	3
Reproductive and environmental variables	3
Variation in breeding demographic structure	5
Spatial synchrony of variation in breeding demographic structure	6
**2. Supporting results**	**8**
Variation in breeding demographic structure and explanatory variables	8
Spatial synchrony of variation in breeding demographic structure	8
**3. Supplementary tables**	**10**
**4. Supplementary figures**	**27**
**References**	**43**

1. Supporting methods

Study systems and data collection

Data used here are from 32 long-term study populations across Europe (Figure 1 – main text), the geographical range of which represents a large part of the species' breeding range (Sullivan *et al.* 2009). The populations occupied various habitat types, predominantly mixed deciduous or evergreen woodlands, with some at more urban sites. The latitudinal range was from Sagunto, Spain (39° 42' N) to Oulu, Finland (65° 05' N), and the longitudinal range was from East Dartmoor, United Kingdom (3° 72' W) to Mayachino, Russia (32° 49' E). Data collection at each site varied slightly, but followed the same general procedure. This involved regular visits to all nest boxes within each study site during the birds' breeding season (approximately April–June) to collect data on first egg date for each breeding attempt (i.e. the date on which the first egg in a brood is laid), the clutch size and the brood size. Additionally, both chicks and parents are individually marked with metal rings that are associated with a unique alphanumeric code, allowing for individual identity to be discerned upon recapture. Upon (re)capture, individuals are aged based on year of hatching if they hatched into the study population, or plumage characteristics if they are immigrants, where subadults (individuals in their first-year) and adults (older than this) can be discriminated based on retention of juvenile feathers in the wing (Svensson 1992).

In total, the study period spanned 1956–2022, collectively consisting of 702 study years and 131,150 captures of 77,964 breeding individuals across the 32 populations. Length of data collection varied between sites (Table S1) depending on the establishment of the long-term study population. Further metadata for all populations can be found through the Studies of Populations of Individual Birds (www.spibirds.org, Culina et al. 2021). Because in the assessed populations almost all great tits first breed as subadults, the number of available nest sites (boxes) exceed the total local population size, and rates of nesting in natural cavities is very low, the monitored breeding populations are a very good proxy for the total number of individuals and therefore the breeding demographic structure in the assessed populations (Dhondt *et al.* 1990; Gamelon *et al.* 2016, 2019; Gosler 1993; Kidd *et al.* 2015; Perrins 1979).

Reproductive and environmental variables

In the main text, we consider the role of reproductive and environmental variables that vary at different spatial scales on temporal variation in, and spatial synchrony of, breeding demographic structure. First, we considered the influence of average clutch size in year $t-1$ on age structure in year t. This was calculated by taking the annual mean number of eggs produced per breeding attempt within each population separately. An individual's clutch size may vary in response to several demographic and environmental factors, such as local population size, predation risk, weather conditions, chick-rearing resource availability and parental quality (Boyce & Perrins 1987; Julliard *et al.* 1997; Møller *et al.* 2020; Perrins 1965; Pettifor *et al.* 2001). We would expect variation

in the within-year mean clutch size to affect the demographic structure of the following annual breeding population, where higher average clutch sizes would lead to greater numbers of recruits (Ahola *et al.* 2009) and therefore a higher proportion of breeding subadults, thus we test this prediction here. Although the number of fledglings may provide a more accurate proxy for recruitment, data on fledgling number were less complete compared to clutch size across the populations, and the number of eggs laid strongly relates to the number of recruits at the pair-level (Perrins 1965; Perrins & Moss 1975).

We also consider the influence of climatic variables on breeding demographic structure: temperature and precipitation, which have both been linked to great tit survival and reproduction (van Balen 1980; Bejer & Rudemo 1985; Bordjan & Tome 2014; Greño *et al.* 2007; Perrins 1965). We extracted daily temperature and precipitation records from a corresponding 0.1° × 0.1° grid cell in the E-OBS dataset version 27.0e for each population (Cornes *et al.* 2018). From these, we assessed the impact of weather on breeding demographic structure by considering weather averages across given time periods (as outlined in the main text). We also considered the influence of the frequency of extreme climatic events (ECEs). Here, we define ECEs as events with an observed occurrence in the extreme 5% of the tail of the relevant distribution across the entire study period (1956–2022) in each population separately (Bailey & van de Pol 2016; Marrot *et al.* 2017; Moreno & Møller 2011), which has been linked to great tit survival and reproduction (Regan & Sheldon 2023). Thus, a 'cold ECE' is defined as when the minimum daily temperature reaches below 5% threshold between June in the year prior to breeding and May in the year of breeding; and a 'hot ECE' as when maximum daily temperature reaches above 95% threshold June–May.

We also considered an environmental variable which varies at a larger spatial scale by using masting data from European beech *Fagus sylvatica*. This variable represents the annual production of beech seeds (Kelly 1994; Silvertown 1980), where maximum production correlates negatively with summer temperatures two years previously, but positively with summer temperatures the year before (Vacchiano *et al.* 2017). Beech masting maintains significant synchrony at spatial scales up to 1500km (Bogdziewicz *et al.* 2021), and its variation has been linked to spatial synchrony of population size in tits (Sæther *et al.* 2007). We obtained data from a long-term continental-scale dataset of masting time series of beech up to 2017 (MASTREE+, Hacket-Pain et al. 2022). For each year of data collection for each breeding population, we extracted a masting value from the year prior to breeding from the site closest to that of the population. Beech masting was measured on an ordinal scale 1–5 where 1 represents lowest reproductive output (Hacket-Pain *et al.* 2022). The central coordinates for masting sites were all less than 1500km from the focal breeding population, which is the spatial scale at which beech masting remains synchronised (Bogdziewicz *et al.* 2021), and most were much closer (median, IQR: 143km, 88–297km). However, as set out in the main text, to assess the influence of beech

masting measured at a more local spatial scale, we created a subset of the data including only annual populations where beech mast data was collected 100km or closer to the breeding site (13 populations, n = 223).

Finally, in addition to analysis outlined in the main text, we considered an environmental variable that varies at a continental scale by assessing the influence of the North Atlantic Oscillation (NAO) index. The NAO index is based on the difference in sea-level pressure between the subtropical high-pressure centre near the Azores and the subpolar low-pressure centre south and east of Greenland, the variation of which is linked to annual fluctuations in temperature and precipitation (Hurrell 1995; Lamb & Peppler 1987; Wanner *et al.* 2001). Generally, positive NAO values during the winter correspond to wetter, warmer weather and earlier springs in northern Europe and drier, warmer weather and advanced springs in southern Europe (Gordo & Sanz 2010; Post & Stenseth 1999). We extracted an annual winter NAO value from The Climate Data Guide (Hurrell & Phillips 2003; Schneider *et al.* 2013).

Variation in breeding demographic structure

In addition to analysis on the proportion of breeding subadults, we also calculated breeding demographic structure through five alternative methods.

First, we calculated the mean breeding population age. As stated in the main text, exact year of hatching is known for all locally-hatched individuals that are ringed at the nest. Although annual immigration rates can be high in the assessed populations (mean, interquartile range: 69.7%, 57.2–88.3%), in many cases individuals are first caught as subadults (mean, IQR: 57.9%, 44.1–81.4%), and thus can be aged accurately. Therefore, exact age was known for 82.5% of 135,967 captures. For the remaining 17.5% of captured individuals (i.e. birds first caught with adult plumage), a minimum age of 2 was assigned, and subsequent age estimates were based on this (15.3% and 20.1% of females and males). This uncertainty does not affect accuracy of calculating our main demographic structure descriptor, because individuals can still be designated as ’subadult’ or ‘adult’ with certainty. For the calculation of mean breeding population age, this assumption may affect accuracy of the demographic structure descriptor. However, given annual mortality rates >50% (Bouwhuis *et al.* 2009; Clobert *et al.* 1988), the assumption is likely to be accurate in the majority of cases. Given that individuals first encountered as adults in the first year of data collection in each population are all assigned an age of 2, the first three years of data collection were removed from each population in the analysis of mean breeding population age such that more accurate individual age estimates were used (n = 637, whereas n = 702 in the main analyses).

Second, we calculated the proportion of senescent individuals in the breeding population. We assume that senescence begins in great tits at 2.8 years (Bouwhuis *et al.* 2009); therefore, due to annual breeding, senescent individuals are defined as those of 3-years or older. Individuals assigned a minimum age of 2 in the first year of data collection which survive to the next year are accurately assigned as ‘senescent individual’ (because they must be 3-years or older). Thus, only the first year of data collection was removed from each population (n = 688).

Finally, we calculated δ_t, the temporal deviations in the described structures (proportion of subadults, mean breeding population age, and proportion of senescent individuals) compared to population-specific moving averages with a window size of 3 years. Specifically, $\delta_t = i_t - (\sum_{t-1}^{t-3} i_t)/3$, where i_t is any of the above structural measures in year *t*. This metric addresses how the explanatory variables might induce momentary change in structure, beyond how those variables correlate with static measures of yearly structure. In order to compare the static age structure measure to a three-year average, data were restricted to populations with at least four years of continuous data. All age structure descriptors were highly correlated (Figure S2).

In addition to testing for associations between reproductive and environmental variables and variation in breeding demographic structure, as in the main text, we also explored overall time trends in variation in breeding demographic structure. We did this by using the same linear mixed-effects model of the form

$$y_{i,j} = \beta_{\text{int}} + u_{\text{int},i} + (\beta_{\text{year}} + u_{\text{year},i})Z_{i,j} + \varepsilon_{i,j}$$

where y is the breeding demographic structure descriptor per population i and year j, β_{int} is an intercept, $u_{\text{int},i}$ denotes random intercepts for each population assumed to have a normal prior distribution with mean 0 and standard deviation $\sigma_{u_{\text{int}}}$, β_{year} is a slope for the linear time trend, $u_{\text{year},i}$ denotes random slopes for the linear time trends of each population also assumed to have a normal prior distribution, Z_{ij} is the time indicator per annual population, and ε_{ij} is the residual error, assumed to have a normal prior distribution.

Spatial synchrony of variation in breeding demographic structure

In addition to the analyses in the main text using the proportion of subadults, we also ran the spatial autocorrelation model (Equation 2) using our five alternative descriptors of breeding demographic structure.

Additionally, in the main text, we use data from annual populations only if the population included at least 20 individuals (mean, IQR: 230, 60–356) and at least 25% of the population was aged (mean, IQR: 56.0%, 36.1–78.2%; n = 702). To assess whether these cut-offs were too lenient and

significantly change results, we also ran our spatial autocorrelation model (Equation 2) using alternative cut-offs with regard to total annual population size and the proportion of aged individuals within the breeding population. Specifically: 20 individuals and 50% aged (n = 597); 20 individuals and 75% aged (n = 276); 30 individuals and 25% aged (n = 682); 30 individuals and 50% aged (n = 582); 30 individuals and 75% aged (n = 269); 50 individuals and 25% aged (n = 638); 50 individuals and 50% aged (n = 546); and 50 individuals and 75% aged (n = 252).

Some research suggests that the degree of spatial synchrony has been increasing over time in natural populations (Koenig & Liebhold 2016). Although this research is primarily concerned with the evidence for, and drivers of, spatial synchrony in breeding demographic structure averaged over the entire study period (1956–2022), we additionally ran our spatial autocorrelation model on a subset of the data (2000–2022) to assess whether there was greater synchrony in more recent times.

Finally, our main analytical framework excludes the possibility of average negative synchrony. However, the parametric approach applied is beneficial when assessing wide-scale spatial synchrony in ecological variables (Bjørnstad *et al.* 1999; Engen *et al.* 2005; Herfindal *et al.* 2020; Vriend *et al.* 2023), as it allows for formal comparisons of synchrony in demographic characteristics by providing estimated parameters, such as the standard deviation (l), which non-parametric methods do not yield (Lande et al. 1999; Grøtan et al. 2005). To explore whether alternative analytical approaches that allow for on average negative correlations greatly affected our results, we applied a Generalised Additive Model (GAM) to model the correlations in breeding demographic structure between paired sites as a function of distance. The correlation in subadult proportion was the response variable, with distance as the predictor. To account for nonlinearity, we incorporated a smoothing function with an adaptive basis, allowing for a flexible fit over the range of distances. The model also applied weights based on the number of overlapping years between paired sites' time series, such that population pairs with more overlapping years had greater influence towards the estimated average (a)synchrony. While this semi-parametric approach produces model predictions for estimated average (a)synchrony in temporal variation in subadult proportion, it does not yield estimated parameters which can be formally compared.

2. Supporting results

Variation in breeding demographic structure and explanatory variables

We found very weak but significant temporal trends in breeding demographic structure. Specifically, we find that over time there was a slight decrease in the proportion of breeding subadults (posterior mode [95% credible intervals]: -0.007 [-0.011, -0.002]) and a slight increase in the breeding population mean age (0.005 [<0.001, 0.010]). We found no significant time trend in the proportion of breeding senescent individuals (0.002 [-0.002, 0.007]); or in temporal deviations in the described structures (proportion of subadults, mean breeding population age, and proportion of senescent individuals) compared to population-specific moving averages (>-0.001 [-0.003, 0.002]; <0.001 [-0.002, 0.003]; <0.001 [-0.001, 0.003], respectively).

We found similar associations between the reproductive and environmental variables with breeding demographic structure whether defining breeding demographic structure as the proportion of breeding subadults (as in the main text; Figure 2), the mean breeding population age (Figure S4; Table S2), the proportion of senescent individuals (Figure S5; Table S2), or changes in these structures compared to a population-specific moving average (Figure S6; Table S2). In addition to the environmental variables assessed in the main text, we found that slightly older breeding populations followed winters with higher NAO values (proportion of subadults posterior mode [95% credible intervals]: -0.115 [-0.189, -0.028]). This may be interpreted similarly to our results found regarding increased winter temperatures, in that when winter NAO values are higher this is associated with warmer winters (see main text discussion).

Spatial synchrony of variation in breeding demographic structure

We found similar patterns of spatial synchrony in our alternative descriptors of breeding demographic structure as was found in the proportion of breeding subadults. The scale of spatial synchrony was greatest in mean breeding population age ($\hat{l}$ = 939km [361km, 1674km]; $\hat{\rho}_{100\text{km}}$ = 0.321 [0.237, 0.407]; $\hat{\rho}_{500\text{km}}$ = 0.279 [0.185, 0.357]; $\hat{\rho}_{2500\text{km}}$ = 0.044 [0.001, 0.183]) and smallest in the proportion of senescent individuals compared to a population-specific 3-year running average ($\hat{l}$ = 478km [243km, 941km]; $\hat{\rho}_{100\text{km}}$ = 0.404 [0.320, 0.488]; $\hat{\rho}_{500\text{km}}$ = 0.261 [0.133, 0.264]; $\hat{\rho}_{2500\text{km}}$ = 0.048 [<0.001, 0.182]). However, in general, patterns of spatial synchrony were very similar across all descriptors of breeding demographic structure, with synchrony decreasing as distance between populations increased (Table S3; Figure S7). This should be expected considering all breeding demographic structure descriptors are highly correlated (Figure S2).

We found similar patterns of spatial synchrony in breeding demographic structure regardless of the cut-off used with respect to which annual populations were included in analyses depending on the total population size and the proportion of the population that was aged (Table S4; Figure S8).

We found similar levels of spatial synchrony in breeding demographic structure when subsetting the data to only include 2000–2022, thus suggesting that there is no evidence of increased spatial synchrony in breeding demographic structure in more recent times (Table S5; Figure S9).

Finally, we found reasonably similar patterns of distance-decay in synchrony when employing a semi-parametric approach allowing for average negative correlations in temporal variation in breeding demographic structure between populations. The model only very weakly predicted on average negative correlation (i.e. asynchrony) at great distances (Table S6; Figure S10).

3. Supplementary tables

Table S1 – The 32 great tit populations used in this study, with information provided for: the location; the population identifier code (used in Figure 1 in the main text, in Figures S1 & S7, and in analysis); initials of data provider(s); latitude (Lat) and longitude (Lon), in decimal degrees; the time series of data used in this study (with total number of years in parentheses); the average within-year population size over the study period (estimated as the number of observed individuals + the number of inferred breeders, where it is assumed there are two individuals per breeding attempt); and the average within-year percentage of aged individuals (calculated as the number of aged individuals divided by the estimated breeding population size, which includes non-captured individuals). Metadata for each population can be found through the Studies of Populations of Individuals Birds (SPI-Birds; www.spibirds.org, Culina et al. 2021).

Location	Population code	Data provider(s)	Lat	Lon	Time series (total years)	Mean pop. size	Mean % aged
Ammersee-Starnbergersee, Germany	AMM	ND	47.5800	11.1400	2011-2019 (9)	597	73.8
Bagley Wood, United Kingdom	BAG	SRE	51.7000	-1.2500	2005-2014 (10)	255	83.7
Balatonfüred, Hungary	BAL	ALi, GS	46.5700	17.5300	2014-2019 (6)	47	69.5
Boshoek, Belgium	BOS	FA, EM	51.0800	4.3200	1994-2018 (25)	465	74.2
Buunderkamp, Netherlands	BUU	MEV	52.0100	5.4500	1984-1992, 1995-2005, 2007-2014 (28)	204	58.6
Can Catà, Spain	CAC	JCS	41.4600	2.1400	2003-2004, 2011, 2013, 2015-2016 (6)	253	28.9
East Dartmoor, United Kingdom	EDM	MDB	50.5900	-3.7200	2014-2015, 2018 (3)	69	30.8
Gotland, Sweden	GOT	LC, BD	57.1000	18.2000	2005-2016, 2021-2022 (14)	889	58.7
Gulya-Domb, Hungary	GUL	ALi, GS	47.0500	17.5300	2019-2022 (4)	61	54.5
Harjavalta, Finland	HAR	TE	61.2000	22.1000	1991-1994 (4)	343	33.9

Hoge Veluwe, Netherlands	HOG	MEV	52.0200	5.5100	1956-2018 (63)	337	76.0
Kilingi-Nõme, Estonia	KIL	ALe	58.1500	24.9600	1972-1992, 1996-2001 (27)	545	65.4
Liesbos, Netherlands	LIE	MEV	51.3500	4.4200	1956-1967, 1971-1974, 1976-2003, 2005-2011, 2014-2018 (56)	116	63.8
Mayachino, Russia	MAY	AA	60.4600	32.4900	1983, 1985, 1990, 2001, 2007-2008, 2013-2016, 2018, 2020-2021 (13)	29	70.4
Montpellier City, France	MON	SPC, AC, AG, ML	43.5900	3.8600	2013-2018 (6)	275	50.8
Mont Ventoux, France	MTV	SPC, AC, AG, ML	44.1000	5.1600	1987-1991, 1993 (6)	48	49.7
Muro, France	MUR	SPC, AC, AG, ML	42.3600	8.5800	2000-2005, 2008, 2012 (8)	42	68.6
Oosterhout, Netherlands	OOS	MEV	51.5200	5.5000	1958, 1964-2018 (56)	100	76.1
Oulu, Finland	OUL	MO, SR, EV	65.0500	25.5300	1970, 1972-1989, 1995, 1999-2021 (43)	266	51.7
Peerdsbos, Belgium	PEE	FA, EM	51.1600	4.2900	1983-2007, 2009-2012, 2014-2018 (36)	230	62.6
Rouvière, France	ROU	SPC, AC, AG, ML	43.4000	3.4000	1992-2006, 2008-2010, 2012-2018 (25)	65	56.7
Sagunto, Spain	SAG	EA, EB	39.4200	-0.1500	1993-2022 (30)	184	86.0
Szentgál, Hungary	SZE	ALi, GS	47.0600	17.4100	2013-2021 (9)	100	61.4
Veszprém, Hungary	VES	ALi, GS	47.0500	17.5400	2013-2022 (10)	104	69.9

Vilma-puszta, Hungary	VIL	ALi, GS	47.0500	17.5200	2014-2022 (9)	42	71.8
Vlieland, Netherlands	VLI	MEV	53.1700	5.0300	1956-2018 (63)	349	79.2
Warnsborn, Netherlands	WAR	KVO	52.0000	5.5200	1983, 1987-2018 (33)	91	71.9
Westerheide, Netherlands	WES	KVO	52.0100	5.5000	1992-2018 (27)	359	67.1
Warsaw Kampinos, Poland	WRSKPN	MS	52.2123	20.4714	2018-2022 (6)	39	62.1
Warsaw Palmiry, Poland	WRSPAL	MS	52.2211	20.4649	2018-2021 (4)	31	63.3
Warsaw Pole Mokotowskie, Poland	WRSPOL	MS	52.1247	21.0698	2017-2022 (6)	53	69.6
Wytham Woods, United Kingdom	WYT	EFC, JAF, BCS, JPW	51.7700	-1.3400	1966-2022 (57)	602	68.0

Table S2 – Associations between temporal variation in breeding demographic structure and 14 reproductive and environmental variables across 32 great tit populations. Results are obtained from linear mixed-effects models of the form

$$y_{i,j} = \beta_{\mathrm{int}} + u_{\mathrm{int},i} + \left(\beta_{\mathrm{expl}} + u_{\mathrm{expl},i}\right)Z_{i,j} + \varepsilon_{i,j} \text{ (Equation 1)}$$

where y is the normalised breeding demographic structure descriptor per population i and year j, β_{int} is an intercept, $u_{\mathrm{int},i}$ denotes random intercepts for each population assumed to have a normal prior distribution with mean 0 and standard deviation $\sigma_{u_{\mathrm{int}}}$, β_{expl} is a slope for the explanatory variable, $u_{\mathrm{expl},i}$ denotes random slopes for the explanatory variable for each population also assumed to have a normal prior distribution, Z_{ij} is the normalised explanatory variable, and ε_{ij} is the residual error, assumed to have a normal prior distribution. The posterior mode denotes the estimated effect size of the normalised explanatory variable on the normalised breeding demographic structure descriptor (β_{expl}), drawn from 12000 posterior samples, with 95% credible intervals.

Breeding demographic structure descriptor	Variable	Posterior mode	95% CrI
Proportion breeding subadults	Clutch size	0.437	[0.310, 0.553]
	Summer temperature	-0.216	[-0.405, -0.038]
	Autumn temperature	-0.091	[-0.276, 0.156]
	Winter temperature	-0.250	[-0.436, -0.053]
	Spring temperature	-0.194	[-0.417, 0.064]
	Summer precipitation	0.065	[-0.042, 0.195]
	Autumn precipitation	-0.010	[-0.091, 0.080]
	Winter precipitation	0.055	[-0.045, 0.168]
	Spring precipitation	-0.068	[-0.146, 0.031]
	Cold ECEs	0.080	[-0.007, 0.161]
	Hot ECEs	-0.101	[-0.186, -0.016]
	Masting	0.225	[0.130, 0.314]
	Masting <100km	0.365	[0.204, 0.538]
	NAO	-0.115	[-0.189, -0.028]
Mean breeding population age	Clutch size	-0.450	[-0.584, -0.323]
	Summer temperature	0.275	[0.068, 0.493]
	Autumn temperature	0.216	[-0.002, 0.418]
	Winter temperature	0.180	[-0.109, 0.414]
	Spring temperature	0.227	[-0.063, 0.500]
	Summer precipitation	-0.078	[-0.214, 0.041]
	Autumn precipitation	0.002	[-0.085, 0.086]
	Winter precipitation	-0.009	[-0.108, 0.093]

	Spring precipitation	0.040	[-0.047, 0.131]
	Cold ECEs	-0.060	[-0.161, 0.051]
	Hot ECEs	0.137	[0.052, 0.230]
	Masting	-0.173	[-0.250, -0.094]
	Masting <100km	-0.248	[-0.519, -0.071]
	NAO	0.094	[0.009, 0.169]
Proportion senescent individuals	Clutch size	-0.390	[-0.496, -0.289]
	Summer temperature	0.173	[-0.001, 0.350]
	Autumn temperature	0.205	[-0.011, 0.407]
	Winter temperature	0.126	[-0.146, 0.343]
	Spring temperature	0.180	[-0.082, 0.425]
	Summer precipitation	-0.026	[-0.140, 0.088]
	Autumn precipitation	-0.005	[-0.086, 0.076]
	Winter precipitation	-0.022	[-0.121, 0.080]
	Spring precipitation	0.031	[-0.070, 0.122]
	Cold ECEs	-0.032	[-0.133, 0.077]
	Hot ECEs	0.098	[0.005, 0.193]
	Masting	-0.081	[-0.159, 0.002]
	Masting <100km	-0.117	[-0.278, 0.046]
	NAO	0.071	[-0.009, 0.151]
Proportion subadults temporal deviations	Clutch size	0.190	[0.086, 0.287]
	Summer temperature	-0.023	[-0.139, 0.079]
	Autumn temperature	-0.009	[-0.150, 0.109]
	Winter temperature	-0.091	[-0.300, 0.020]
	Spring temperature	-0.035	[-0.150, 0.073]
	Summer precipitation	0.061	[-0.034, 0.191]
	Autumn precipitation	-0.018	[-0.107, 0.078]
	Winter precipitation	0.014	[-0.075, 0.119]
	Spring precipitation	-0.046	[-0.137, 0.050]
	Cold ECEs	0.056	[-0.063, 0.150]
	Hot ECEs	-0.058	[-0.156, 0.031]
	Masting	0.284	[0.162, 0.385]
	Masting <100km	0.480	[0.289, 0.766]
	NAO	-0.153	[-0.252, -0.058]
Mean age temporal deviations	Clutch size	-0.215	[-0.342, -0.101]
	Summer temperature	-0.002	[-0.147, 0.235]
	Autumn temperature	0.017	[-0.107, 0.223]
	Winter temperature	0.114	[-0.017, 0.507]
	Spring temperature	0.027	[-0.098, 0.193]
	Summer precipitation	-0.060	[-0.195, 0.049]
	Autumn precipitation	0.007	[-0.085, 0.116]
	Winter precipitation	0.028	[-0.097, 0.141]
	Spring precipitation	0.074	[-0.018, 0.182]

	Cold ECEs	-0.037	[-0.169, 0.133]
	Hot ECEs	0.064	[-0.046, 0.194]
	Masting	-0.250	[-0.362, -0.139]
	Masting <100km	-0.365	[-0.590, -0.149]
	NAO	0.109	[0.008, 0.234]
Proportion senescents temporal deviations	Clutch size	-0.183	[-0.358, -0.078]
	Summer temperature	0.091	[-0.035, 0.288]
	Autumn temperature	0.036	[-0.078, 0.276]
	Winter temperature	0.060	[-0.064, 0.251]
	Spring temperature	0.060	[-0.042, 0.231]
	Summer precipitation	-0.061	[-0.184, 0.035]
	Autumn precipitation	0.014	[-0.078, 0.109]
	Winter precipitation	-0.055	[-0.196, 0.058]
	Spring precipitation	0.037	[-0.061, 0.131]
	Cold ECEs	-0.005	[-0.170, 0.231]
	Hot ECEs	0.096	[-0.002, 0.202]
	Masting	-0.096	[-0.201, 0.016]
	Masting <100km	-0.203	[-0.46, 0.000]
	NAO	0.125	[-0.027, 0.244]

Table S3 – Spatial synchrony of temporal variation in breeding demographic structure in great tit populations. Breeding demographic structure is defined either as the proportion of the breeding population consisting of subadults, the mean breeding population age, the proportion of the breeding population consisting of senescent individuals, and temporal deviations in these described structures (proportion of subadults, mean breeding population age, and proportion of senescent individuals) compared to population-specific moving averages with a window size of 3 years (supporting methods). Estimates are provided for spatial synchrony parameters (calculated in Equation 2 in the main text); and for synchrony at distances of 100km, 500km, 1000km and 2500km.

**Breeding demographic structure descriptor**	**Parameter**	**Median**	**95% credible intervals**
Proportion breeding subadults	ρ_0	0.344	[0.264, 0.424]
	ρ_∞	<0.001	[<0.001, 0.112]
	l	641km	[371km, 1000km]
	$\rho_{100\text{km}}$	0.340	[0.260, 0.416]
	$\rho_{500\text{km}}$	0.253	[0.163, 0.330]
	$\rho_{1000\text{km}}$	0.115	[0.035, 0.205]
	$\rho_{2500\text{km}}$	0.004	[<0.001, 0.112]
Mean breeding population age	ρ_0	0.323	[0.238, 0.411]
	ρ_∞	<0.001	[<0.001, 0.178]
	l	939km	[361km, 1674km]
	$\rho_{100\text{km}}$	0.321	[0.237, 0.407]
	$\rho_{500\text{km}}$	0.279	[0.185, 0.357]
	$\rho_{1000\text{km}}$	0.198	[0.089, 0.287]
	$\rho_{2500\text{km}}$	0.044	[0.001, 0.183]
Proportion senescent individuals	ρ_0	0.334	[0.257, 0.417]
	ρ_∞	<0.001	[<0.001, 0.149]
	l	821km	[407km, 1345km]

	ρ_{100km}	0.331	[0.255, 0.413]
	ρ_{500km}	0.277	[0.192, 0.351]
	ρ_{1000km}	0.172	[0.075, 0.264]
	ρ_{2500km}	0.022	[<0.001, 0.151]
Proportion subadults temporal deviations	ρ_0	0.398	[0.313, 0.480]
	ρ_∞	<0.001	[<0.001, 0.160]
	l	716km	[365km, 1155km]
	ρ_{100km}	0.394	[0.309, 0.473]
	ρ_{500km}	0.312	[0.209, 0.388]
	ρ_{1000km}	0.168	[0.053, 0.276]
	ρ_{2500km}	0.012	[<0.001, 0.161]
Mean age temporal deviations	ρ_0	0.415	[0.328, 0.497]
	ρ_∞	0.009	[<0.001, 0.213]
	l	842km	[391km, 1477km]
	ρ_{100km}	0.412	[0.325, 0.493]
	ρ_{500km}	0.349	[0.256, 0.427]
	ρ_{1000km}	0.227	[0.114, 0.332]
	ρ_{2500km}	0.045	[0.001, 0.215]
Proportion senescents temporal deviations	ρ_0	0.413	[0.326, 0.501]
	ρ_∞	0.047	[<0.001, 0.182]
	l	478km	[243km, 941km]
	ρ_{100km}	0.404	[0.320, 0.488]
	ρ_{500km}	0.261	[0.133, 0.264]
	ρ_{1000km}	0.107	[0.018, 0.235]

	ρ_{2500km}	0.048	[<0.001, 0.182]

Table S4 – Spatial synchrony of temporal variation in breeding demographic structure (proportion of subadults) in great tit breeding populations across different subsets of data depending on the cut-off introduced with respect to the total annual breeding population size and the proportion of the population that was aged. Estimates are provided for spatial synchrony parameters (calculated in Equation 2 in the main text); and for synchrony at distances of 100km, 500km, 1000km and 2500km. The bolded cells refer to the population size and proportion aged cut-off used in the main analyses and text. As can be seen, the parameters obtained across the datasets are very similar to one another (Figure S8).

Population size cut-off	Proportion of the population aged cut-off	n	Parameter	Median	95% credible intervals
**20**	**25%**	**702**	$\boldsymbol{\rho_0}$	**0.344**	**[0.264, 0.424]**
			$\boldsymbol{\rho_\infty}$	**<0.001**	**[<0.001, 0.112]**
			$\boldsymbol{l}$	**641km**	**[371km, 1000km]**
			$\boldsymbol{\rho_{100km}}$	**0.340**	**[0.260, 0.416]**
			$\boldsymbol{\rho_{500km}}$	**0.253**	**[0.163, 0.330]**
			$\boldsymbol{\rho_{1000km}}$	**0.115**	**[0.035, 0.205]**
			$\boldsymbol{\rho_{2500km}}$	**0.004**	**[<0.001, 0.112]**
20	50%	597	ρ_0	0.431	[0.345, 0.515]
			ρ_∞	<0.001	[<0.001, 0.133]
			l	561km	[339km, 857km]
			ρ_{100km}	0.423	[0.339, 0.502]
			ρ_{500km}	0.292	[0.183, 0.376]
			ρ_{1000km}	0.109	[0.025, 0.224]
			ρ_{2500km}	0.002	[<0.001, 0.133]
20	75%	276	ρ_0	0.511	[0.377, 0.637]
			ρ_∞	0.068	[<0.001, 0.309]
			l	589km	[160km, 1556km]

			ρ_{100km}	0.500	[0.374, 0.608]
			ρ_{500km}	0.374	[0.182, 0.501]
			ρ_{1000km}	0.218	[0.033, 0.399]
			ρ_{2500km}	0.086	[<0.001, 0.316]
30	25%	682	ρ_0	0.333	[0.250, 0.415]
			ρ_∞	<0.001	[<0.001, 0.125]
			l	643km	[346km, 1031km]
			ρ_{100km}	0.328	[0.248, 0.408]
			ρ_{500km}	0.247	[0.153, 0.320]
			ρ_{1000km}	0.114	[0.031, 0.213]
			ρ_{2500km}	0.006	[<0.001, 0.126]
30	50%	582	ρ_0	0.439	[0.353, 0.519]
			ρ_∞	<0.001	[<0.001, 0.150]
			l	595km	[343km, 906km]
			ρ_{100km}	0.432	[0.348, 0.508]
			ρ_{500km}	0.310	[0.194, 0.394]
			ρ_{1000km}	0.126	[0.031, 0.244]
			ρ_{2500km}	0.003	[<0.001, 0.150]
30	75%	269	ρ_0	0.514	[0.386, 0.652]
			ρ_∞	0.051	[<0.001, 0.322]
			l	610km	[171km, 1600km]
			ρ_{100km}	0.505	[0.379, 0.621]
			ρ_{500km}	0.383	[0.181, 0.509]
			ρ_{1000km}	0.225	[0.038, 0.404]

			ρ_{2500km}	0.077	[<0.001, 0.327]
50	25%	638	ρ_0	0.350	[0.269, 0.435]
			ρ_∞	<0.001	[<0.001, 0.129]
			l	703km	[365km, 1132km]
			ρ_{100km}	0.346	[0.267, 0.427]
			ρ_{500km}	0.272	[0.169, 0.348]
			ρ_{1000km}	0.143	[0.043, 0.239]
			ρ_{2500km}	0.010	[<0.001, 0.131]
50	50%	546	ρ_0	0.421	[0.334, 0.501]
			ρ_∞	<0.001	[<0.001, 0.160]
			l	679km	[358km, 1111km]
			ρ_{100km}	0.416	[0.330, 0.492]
			ρ_{500km}	0.323	[0.206, 0.402]
			ρ_{1000km}	0.162	[0.048, 0.280]
			ρ_{2500km}	0.009	[<0.001, 0.161]
50	75%	252	ρ_0	0.472	[0.339, 0.618]
			ρ_∞	<0.001	[<0.001, 0.288]
			l	697km	[193km, 1780km]
			ρ_{100km}	0.466	[0.335, 0.592]
			ρ_{500km}	0.361	[0.171, 0.487]
			ρ_{1000km}	0.211	[0.031, 0.386]
			ρ_{2500km}	NA	NA

Table S5 – Spatial synchrony of temporal variation in breeding demographic structure (proportion of subadults) in great tit populations across a subset of the data 2000–2022. Estimates are provided for spatial synchrony parameters (calculated in Equation 2 in the main text); and for synchrony at distances of 100km, 500km, 1000km and 2500km. As can be seen, the parameters obtained from analysis are very similar to when synchrony is assessed across all data (Figure S9).

**Parameter**	**Median**	**95% credible intervals**
ρ_0	0.332	[0.218, 0.445]
ρ_∞	<0.001	[<0.001, 0.157]
l	672km	[295km, 1266km]
$\rho_{100\mathrm{km}}$	0.327	[0.213, 0.438]
$\rho_{500\mathrm{km}}$	0.251	[0.128, 0.354]
$\rho_{1000\mathrm{km}}$	0.127	[0.032, 0.246]
$\rho_{2500\mathrm{km}}$	0.010	[<0.001, 0.154]

Table S6 – Spatial synchrony of breeding demographic structure (subadult proportion) using a semi-parametric approach allowing for on average negative synchrony. Results were obtained from a Generalised Additive Model (GAM) that modelled the correlations in breeding demographic structure between paired sites as a function of distance. The correlation in subadult proportion was the response variable, with distance as the predictor, and a smoothing function with an adaptive basis allowing for a flexible fit over the range of distances. The model also applied weights based on the number of overlapping years between paired sites' time series, such that population pairs with more overlapping years greater had greater influence towards the estimated average (a)synchrony. Estimates are provided predicted values of the average correlation in temporal variation in breeding demographic structure between populations obtained from the GAM; and for spatial synchrony parameters obtained from our main approach (calculated in Equation 2 in the main text) at the same distances. As can be seen, the predicted correlations through the semi-parametric approach and the parameters obtained from our main framework are relatively similar, showing similar distance-decay in spatial synchrony, and the GAM only predicts on average negative synchrony very weakly at great distances (Figure S10).

**Distance (km)**	**GAM predicted value [95% Conf. Int.]**	**$\hat{\rho}$ from main approach [95% Cred. Int]**
50	0.293 [0.200, 0.386]	0.344 [0.264, 0.422]
100	0.249 [0.176, 0.321]	0.341 [0.260, 0.417]
250	0.266 [0.185, 0.347]	0.318 [0.239, 0.390]
500	0.248 [0.182, 0.315]	0.254 [0.163, 0.330]
1000	0.112 [0.059, 0.165]	0.115 [0.034, 0.205]
2500	-0.125 [-0.228, -0.022]	0.004 [<0.001, 0.112]

Table S7 – Spatial synchrony of temporal variation in the proportion of subadults in great tit breeding populations after accounting for variation in reproductive and environmental variables. Estimates are provided for spatial synchrony parameters (calculated in Equation 2 in the main text); and for synchrony at distances of 100km, 500km, 1000km and 2500km.

Variable accounted for	Parameter	Median	95% CrI
Clutch size	ρ_0	0.302	[0.215, 0.392]
	ρ_∞	<0.001	[<0.001, 0.111]
	l	564km	[267km, 964km]
	$\rho_{100\text{km}}$	0.297	[0.212, 0.383]
	$\rho_{500\text{km}}$	0.201	[0.092, 0.282]
	$\rho_{1000\text{km}}$	0.075	[0.009, 0.175]
	$\rho_{2500\text{km}}$	0.002	[<0.001, 0.112]
Summer temperature	ρ_0	0.348	[0.262, 0.433]
	ρ_∞	<0.001	[<0.001, 0.127]
	l	618km	[314km, 1018km]
	$\rho_{100\text{km}}$	0.343	[0.258, 0.425]
	$\rho_{500\text{km}}$	0.250	[0.147, 0.330]
	$\rho_{1000\text{km}}$	0.110	[0.025, 0.215]
	$\rho_{2500\text{km}}$	0.004	[<0.001, 0.127]
Autumn temperature	ρ_0	0.339	[0.251, 0.431]
	ρ_∞	<0.001	[<0.001, 0.119]
	l	646km	[334km, 1046km]
	$\rho_{100\text{km}}$	0.335	[0.248, 0.423]
	$\rho_{500\text{km}}$	0.251	[0.153, 0.332]
	$\rho_{1000\text{km}}$	0.118	[0.032, 0.220]
	$\rho_{2500\text{km}}$	0.006	[<0.001, 0.119]
Winter temperature	ρ_0	0.326	[0.231, 0.410]
	ρ_∞	<0.001	[<0.001, 0.128]
	l	709km	[332km, 1199km]
	$\rho_{100\text{km}}$	0.322	[0.229, 0.402]
	$\rho_{500\text{km}}$	0.251	[0.149, 0.324]
	$\rho_{1000\text{km}}$	0.133	[0.040, 0.231]
	$\rho_{2500\text{km}}$	0.010	[<0.001, 0.130]
Spring temperature	ρ_0	0.344	[0.262, 0.430]
	ρ_∞	<0.001	[<0.001, 0.146]
	l	693km	[357km, 1107km]
	$\rho_{100\text{km}}$	0.340	[0.259, 0.424]
	$\rho_{500\text{km}}$	0.265	[0.168, 0.343]
	$\rho_{1000\text{km}}$	0.139	[0.043, 0.233]
	$\rho_{2500\text{km}}$	0.010	[<0.001, 0.147]
Summer precipitation	ρ_0	0.335	[0.248, 0.421]

	ρ_{∞}	<0.001	[<0.001, 0.126]
	l	672km	[339km, 1118km]
	ρ_{100km}	0.332	[0.246, 0.414]
	ρ_{500km}	0.254	[0.156, 0.332]
	ρ_{1000km}	0.127	[0.033, 0.230]
	ρ_{2500km}	0.007	[<0.001, 0.127]
Autumn precipitation	ρ_{0}	0.323	[0.241, 0.412]
	ρ_{∞}	<0.001	[<0.001, 0.119]
	l	612km	[310km, 1014km]
	ρ_{100km}	0.319	[0.239, 0.403]
	ρ_{500km}	0.233	[0.129, 0.314]
	ρ_{1000km}	0.107	[0.022, 0.204]
	ρ_{2500km}	0.004	[<0.001, 0.119]
Winter precipitation	ρ_{0}	0.347	[0.258, 0.433]
	ρ_{∞}	<0.001	[<0.001, 0.110]
	l	604km	[330km, 997km]
	ρ_{100km}	0.341	[0.255, 0.426]
	ρ_{500km}	0.247	[0.247, 0.328]
	ρ_{1000km}	0.104	[0.104, 0.211]
	ρ_{2500km}	0.003	[0.003, 0.111]
Spring precipitation	ρ_{0}	0.348	[0.259, 0.432]
	ρ_{∞}	<0.001	[<0.001, 0.133]
	l	662km	[338km, 1101km]
	ρ_{100km}	0.343	[0.257, 0.426]
	ρ_{500km}	0.260	[0.159, 0.335]
	ρ_{1000km}	0.127	[0.035, 0.228]
	ρ_{2500km}	0.006	[<0.001, 0.133]
Cold ECEs	ρ_{0}	0.327	[0.242, 0.415]
	ρ_{∞}	<0.001	[<0.001, 0.118]
	l	675km	[325km, 1148km]
	ρ_{100km}	0.323	[0.239, 0.410]
	ρ_{500km}	0.247	[0.146, 0.328]
	ρ_{1000km}	0.122	[0.033, 0.220]
	ρ_{2500km}	0.007	[<0.001, 0.120]
Hot ECEs	ρ_{0}	0.347	[0.264, 0.432]
	ρ_{∞}	<0.001	[<0.001, 0.125]
	l	632km	[343km, 1001km]
	ρ_{100km}	0.342	[0.260, 0.425]
	ρ_{500km}	0.254	[0.153, 0.334]
	ρ_{1000km}	0.117	[0.028, 0.215]
	ρ_{2500km}	0.005	[<0.001, 0.125]
Masting	ρ_{0}	0.272	[0.185, 0.362]
	ρ_{∞}	<0.001	[<0.001, 0.134]
	l	793km	[324km, 1440km]

	ρ_{100km}	0.269	[0.183, 0.357]
	ρ_{500km}	0.221	[0.132, 0.295]
	ρ_{1000km}	0.136	[0.037, 0.220]
	ρ_{2500km}	0.019	[<0.001, 0.137]
Masting < 100km	ρ_0	0.301	[0.104, 0.573]
	ρ_∞	<0.001	[<0.001, 0.261]
	l	255km	[19km, 437924km]
	ρ_{100km}	0.239	[0.030, 0.419]
	ρ_{500km}	0.109	[<0.001, 0.303]
	ρ_{1000km}	NA	NA
	ρ_{2500km}	NA	NA
NAO	ρ_0	0.315	[0.228, 0.402]
	ρ_∞	<0.001	[<0.001, 0.119]
	l	697km	[329km, 1220km]
	ρ_{100km}	0.311	[0.226, 0.394]
	ρ_{500km}	0.240	[0.140, 0.319]
	ρ_{1000km}	0.127	[0.032, 0.227]
	ρ_{2500km}	0.009	[<0.001, 0.120]

4. Supplementary figures

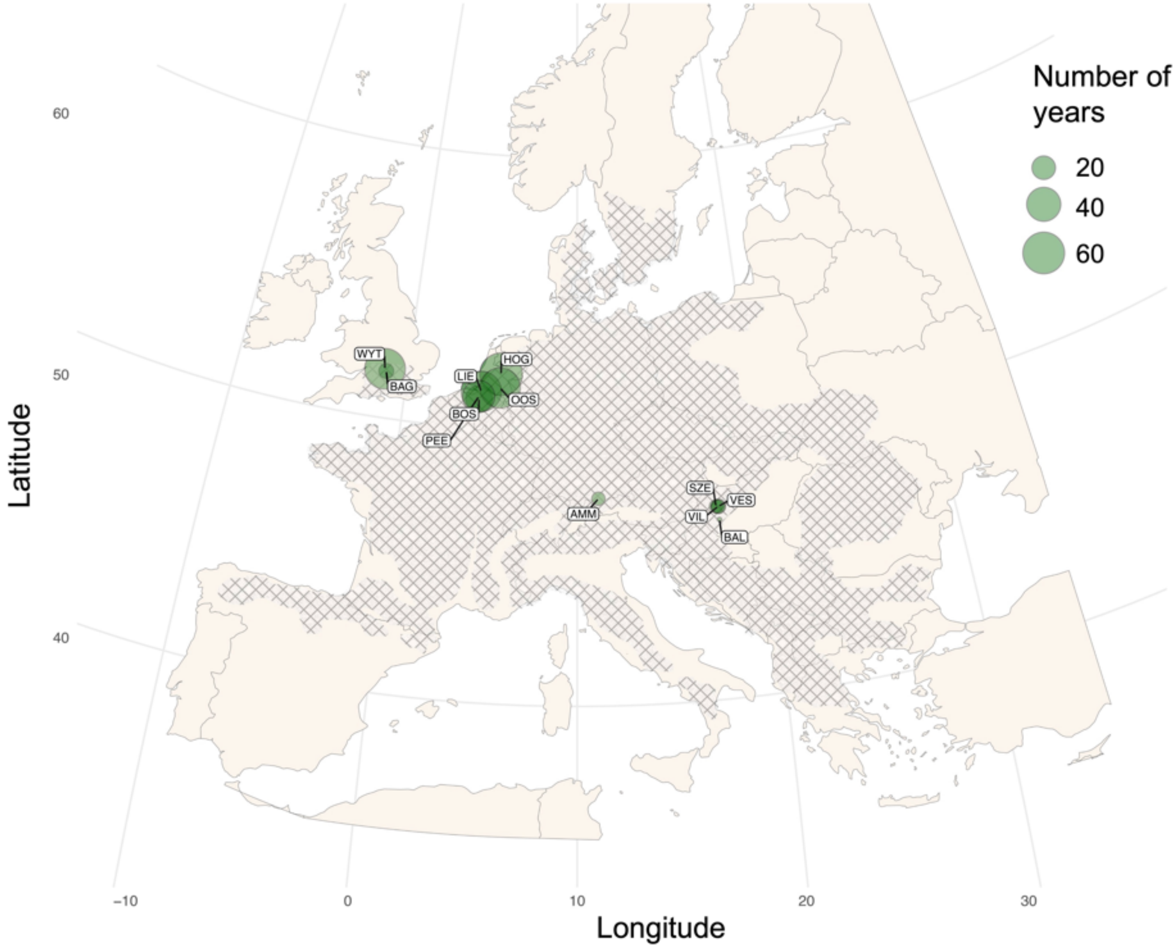

Figure S1 – Map of the 12 great tit study populations across Europe included in our sub-analysis assessing the influence of masting at a more local spatial scale. The annual populations include those that are within the continuous distribution range of beech (shown approximately in cross-hatching on the map, adapted from Bolte et al. 2007) and where the beech data was collected within 100km of the populations. Dark green points represent the great tit populations, with point size relative to the number of years in the time series.

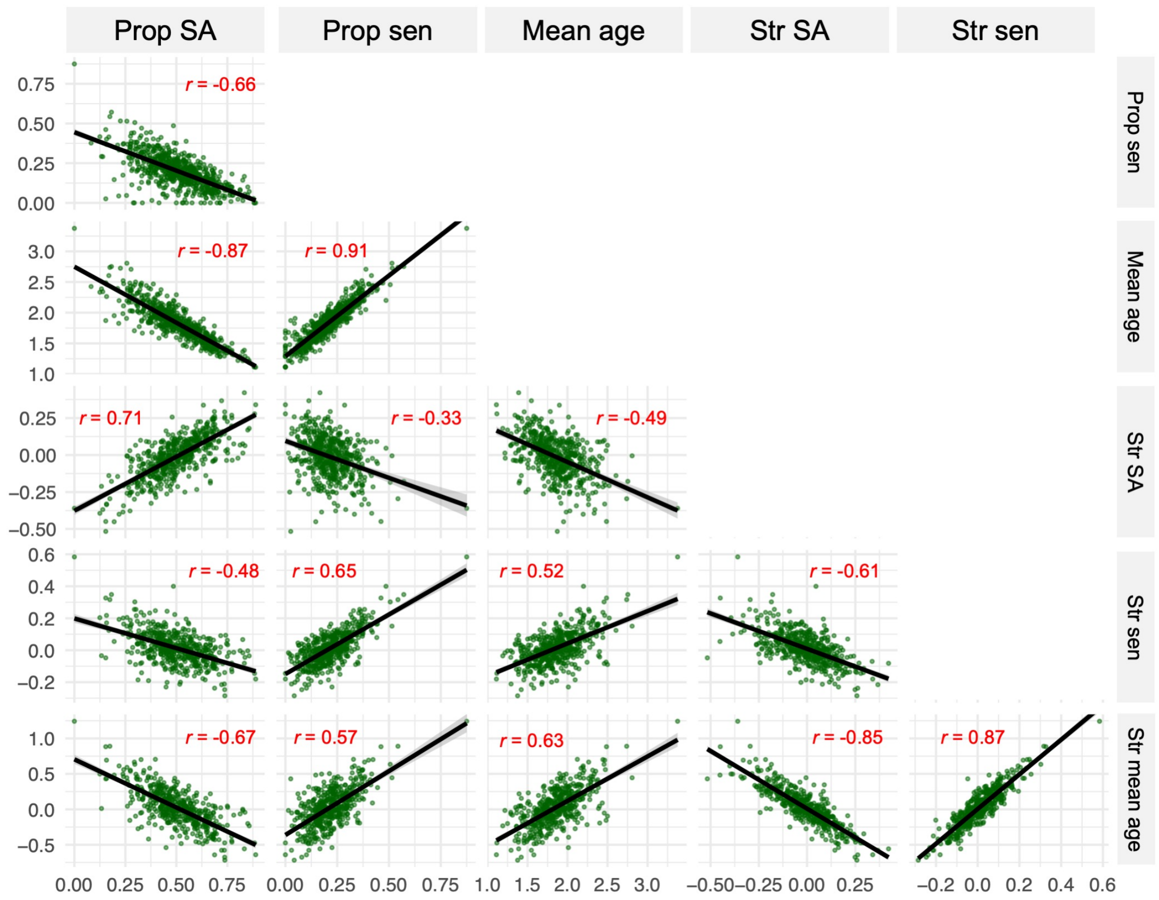

Figure S2 – Correlations between the different breeding demographic structure descriptors (“Prop SA” = proportion of breeding population consisting of subadults, “Prop sen” = proportion of breeding population consisting of senescent individuals, “Mean age” = mean breeding population age, “Str SA” = temporal deviation of the proportion of subadults compared to a population-specific moving averages with a window size of 3 years, “Str sen” = temporal deviation of the proportion of senescent individuals compared to a population-specific moving average, ”Str mean age” = temporal deviation of the mean breeding population age compared to a population-specific moving average) used in this study. In all plots, the black line is a linear regression which models the relationship between the two raw breeding demographic structure measures (labelled on each of the x and y axis), and the shading around this shows the 95% confidence intervals. The relationship is significant where $p < 0.05$ in all cases, and the r value obtained from a Spearman’s rank correlation labelled in red on all plots.

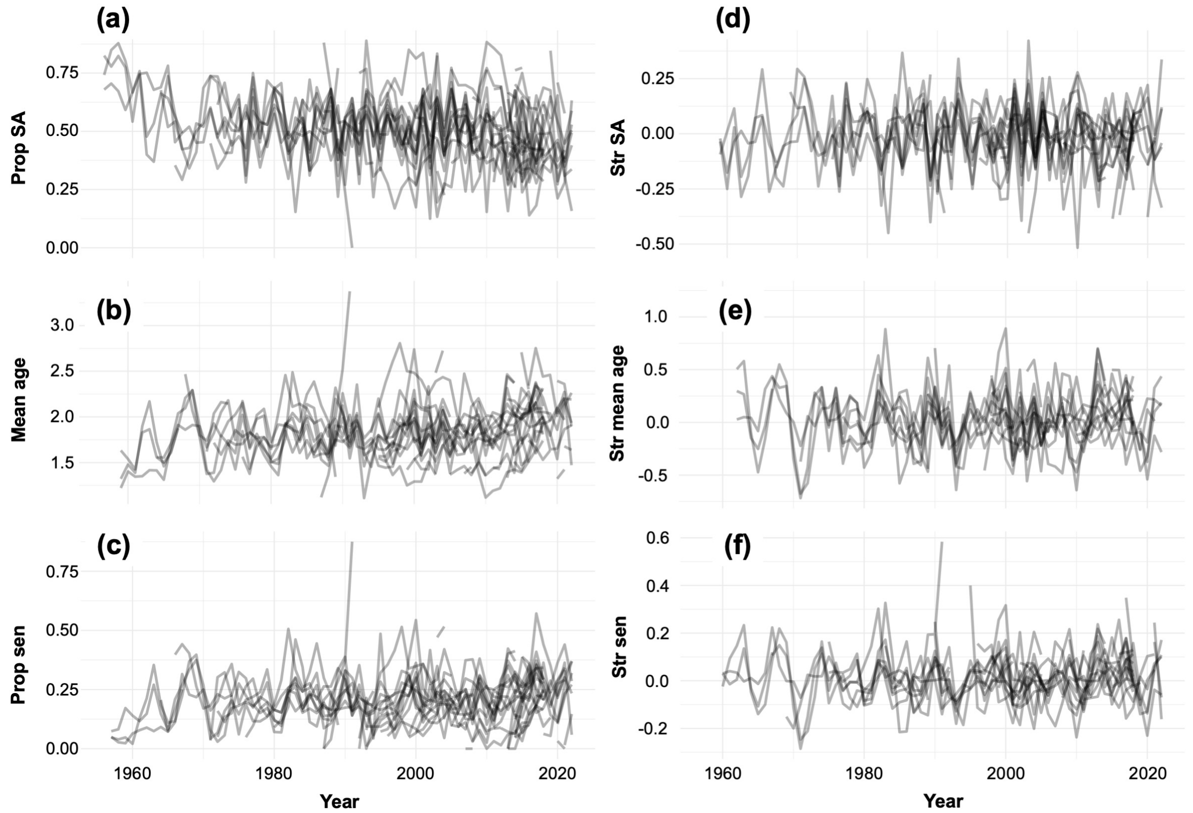

Figure S3 – Temporal variation in breeding demographic structure across European great tit populations. In all plots and analysis, annual populations were only included if the population included at least 20 individuals (mean, IQR: 230, 60–356) and at least 25% of the population was aged (mean, IQR: 56.0%, 36.1–78.2%). Each line corresponds to a continuous time series from a single population (i.e. broken lines represent time series where some annual breeding populations consisted of either less than 20 individuals or less than 25% aged individuals). (a) Shows the proportion of breeding subadults (32 populations, n = 702); (b) mean breeding population age (32 populations, n = 637); (c) proportion of breeding senescent individuals (32 populations, n = 688); (d) change in the proportion of subadults compared to a 3-year running mean (30 populations, n = 549); (e) change in the mean breeding population age compared to a 3-year running mean (25 populations, n = 493); and (f) change in the proportion of senescent individuals compared to a running mean (27 populations, n = 536). Note that the striking synchrony across populations in the earliest years of the time series is likely due to the longest-running population studies being both large (hence less subject to sampling error) and relatively close in space.

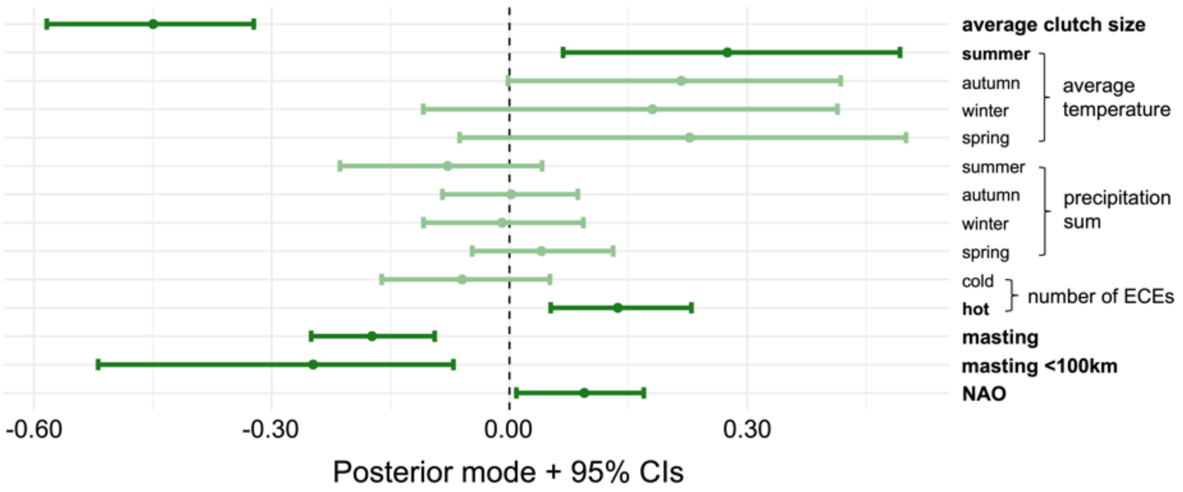

Figure S4 – Posterior modes obtained from linear mixed-effects models which analyse the association between temporal variation in mean breeding population age and 14 reproductive and environmental variables across 32 great tit populations. Each point represents the fixed-effect slope (β_{expl} in Equation 1) for a specific predictor variable (on the y-axis), and error bars denote 95% credible intervals. Points and error bars are reduced in saturation when credible intervals overlap zero, and explanatory variable text is bolded when they do not.

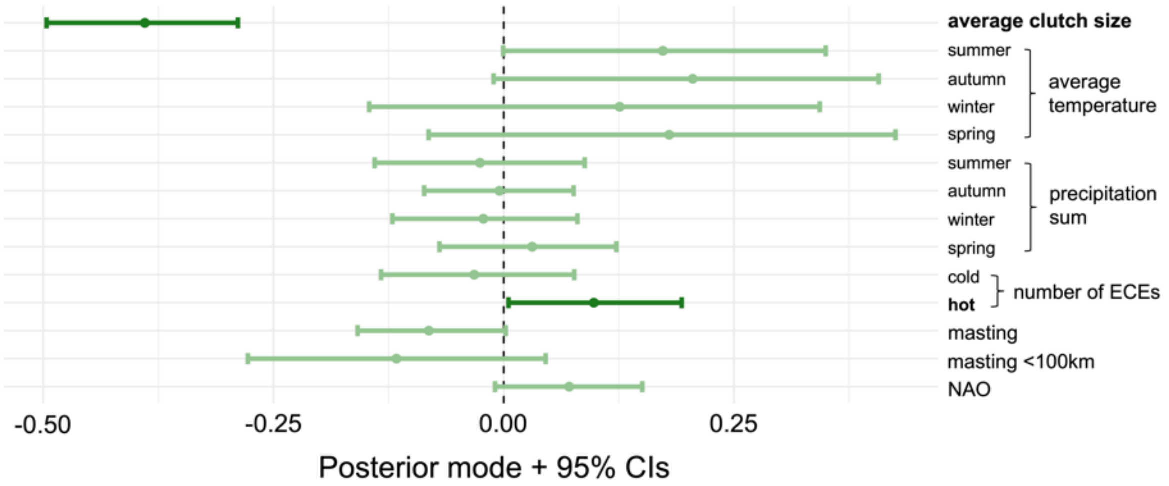

Figure S5 – Posterior modes obtained from linear mixed-effects models which analyse the association between temporal variation in the proportion of breeding senescent individuals and 14 reproductive and environmental variables across 32 great tit populations. Each point represents the fixed-effect slope (β_{expl} in Equation 1) for a specific predictor variable (on the y-axis), and error bars denote 95% credible intervals. Points and error bars are reduced in saturation when credible intervals overlap zero, and explanatory variable text is bolded when they do not.

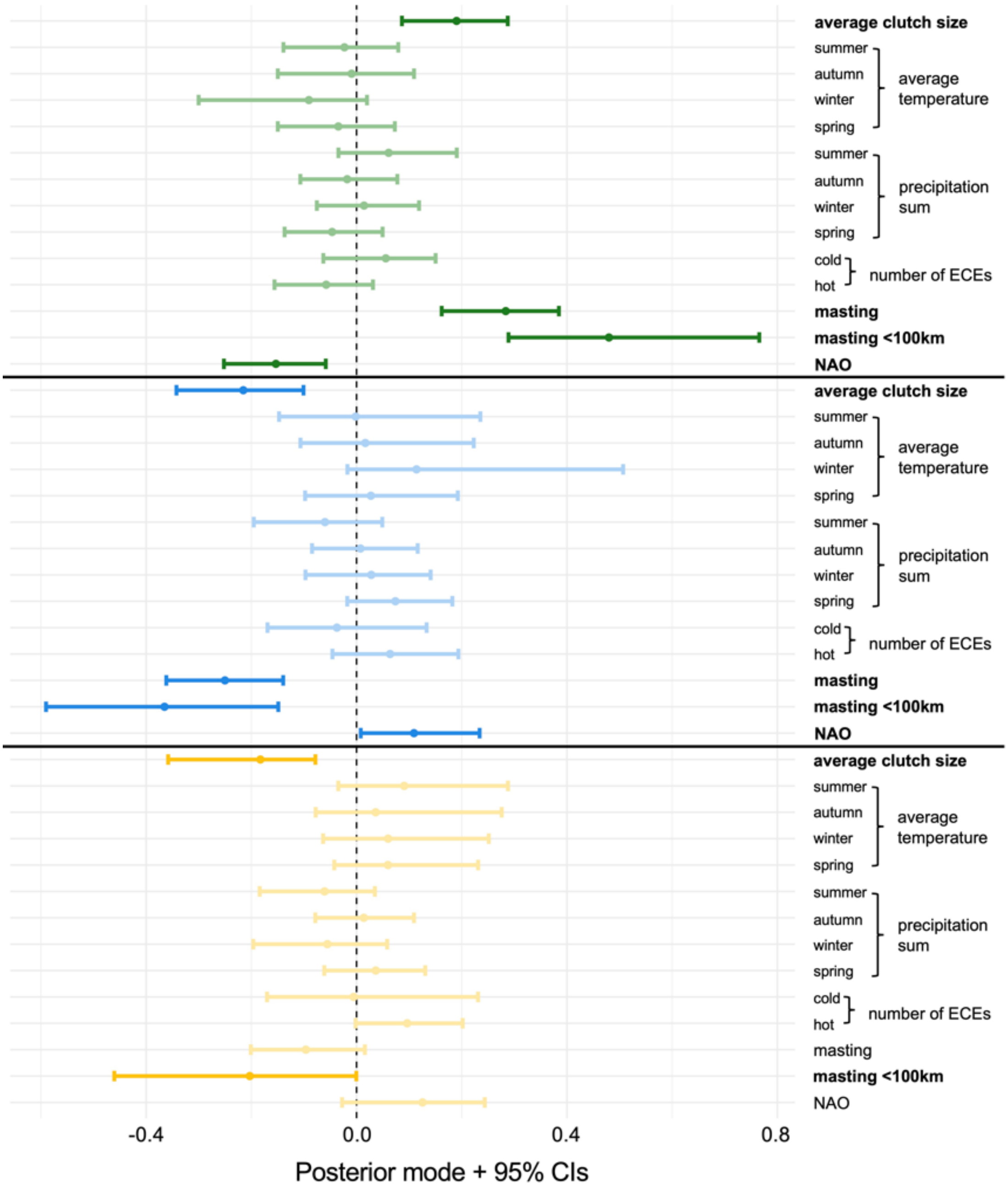

Figure S6 – Posterior modes obtained from linear mixed-effects models which analyse the association between temporal variation in breeding demographic structure (defined as the difference between the within-year static breeding demographic structure measure and that of a running average calculated as the mean in the three years previous) and 14 reproductive and environmental variables across 32 great tit populations. Each point represents the fixed-effect slope (β_{expl} in Equation 1) for a specific predictor variable (on the y-axis), and error bars denote 95% credible intervals. Points and error bars are reduced in saturation when credible intervals overlap zero, and explanatory variable text is bolded when they do not. Green points are from analysis assessing the proportion of breeding subadults, blue for the mean breeding population age, and yellow for the proportion of senescent individuals.

429

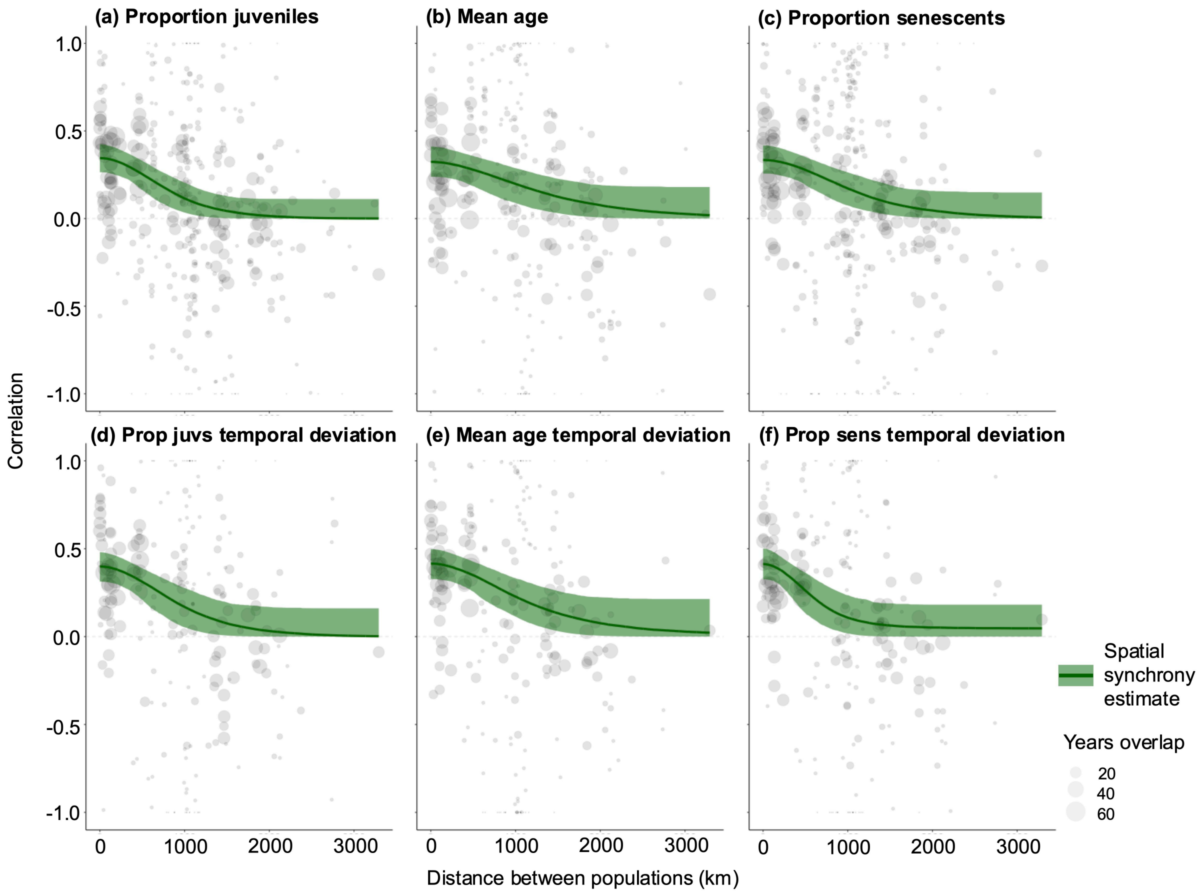

Figure S7 – Spatial synchrony of temporal variation in breeding demographic structure across great tit populations in relation to the distance between them. In all plots, distance between populations (km) is on the x-axis and correlation between paired sites is on the y-axis. (a) Shows spatial synchrony of temporal fluctuations in the proportion of subadults, where the green line is the median estimate of spatial synchrony (calculated in Equation 2) based on 2000 bootstrap replicates, with light green shading representing 95% credible intervals, and point size relative to the number of years of overlap between the time series of pairwise sites. (b) Shows spatial synchrony in the mean breeding population age; (c) shows spatial synchrony in the proportion of the breeding population consisting of senescent individuals; and (d–f) shows spatial synchrony in the temporal deviations in these structures, respectively, compared to a population-specific moving average with a window size of 3 years.

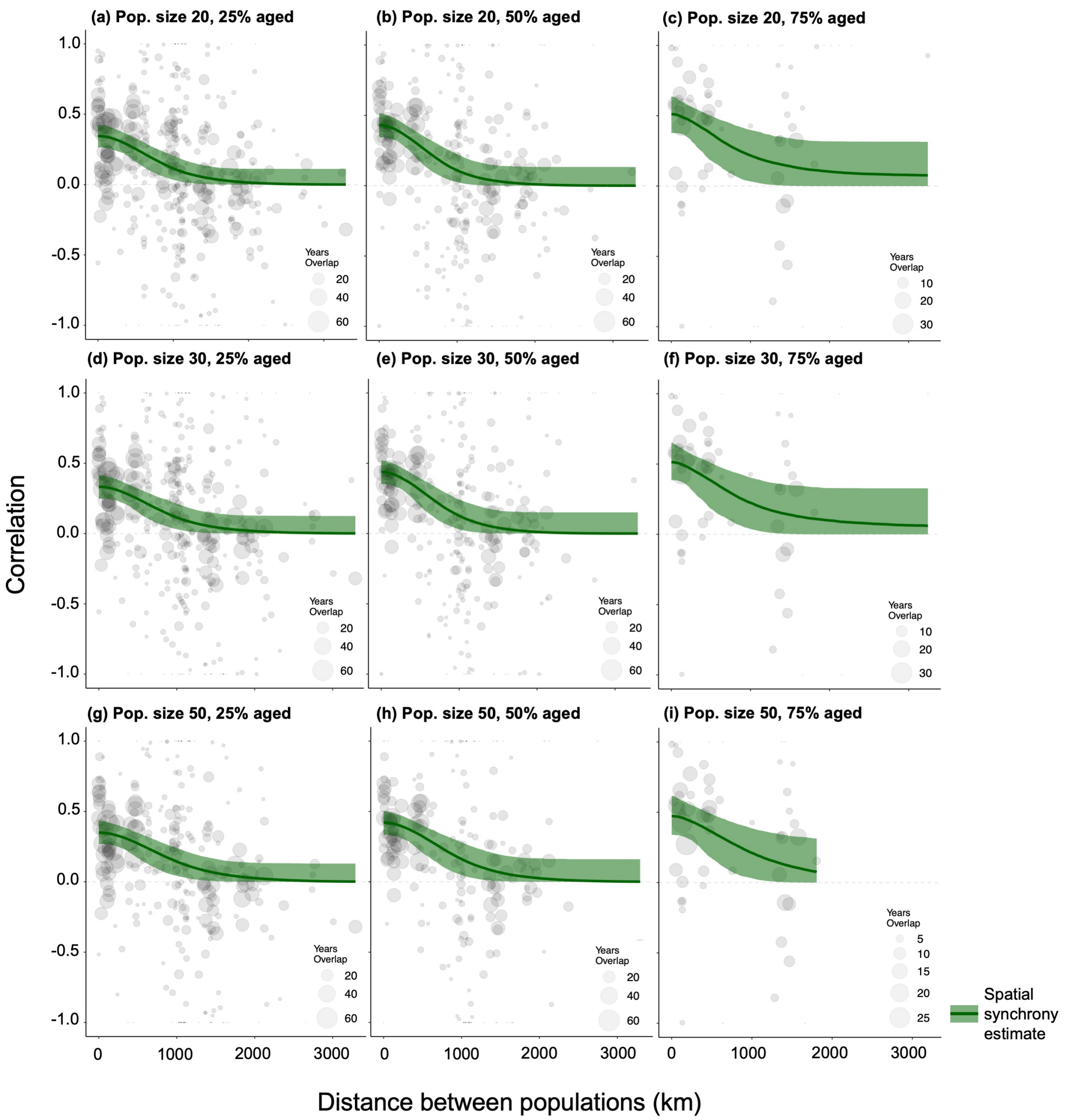

Figure S8 – Spatial synchrony of temporal variation in breeding demographic structure (proportion of subadults) in great tit breeding populations across different subsets of data depending on the cut-off introduced with respect to the total annual breeding population size and the proportion of the population that was aged. In all plots, the green line is the median estimate of spatial synchrony (calculated in Equation 2) based on 2000 bootstrap replicates, with light green shading representing 95% credible intervals. As can be seen, the estimated pattern of spatial synchrony is similar regardless of the cut-off chosen.

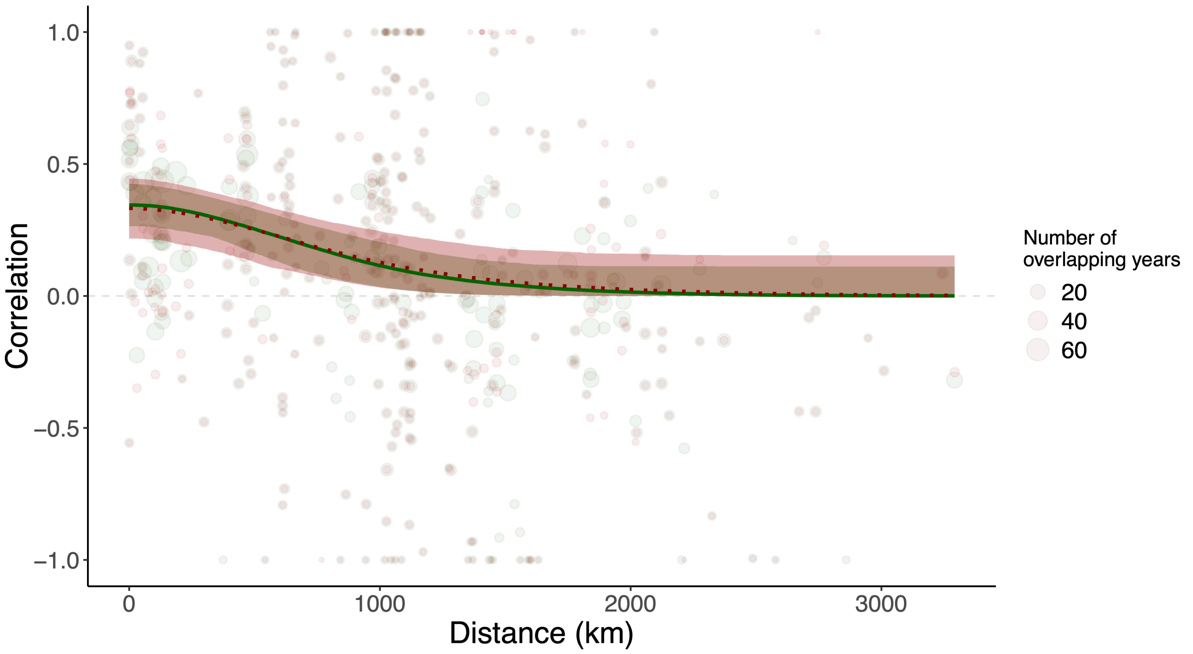

Figure S9 – Spatial synchrony of temporal variation in breeding demographic structure (proportion of subadults) in great tit populations across all data 1956–2022 (green) and a subset of the data 2000–2022 (red). Distance between populations (km) is on the x-axis and correlation between paired sites is on the y-axis. As can be seen, the observed degree of spatial synchrony is very similar across all time, and on a subset of the data in more recent time.

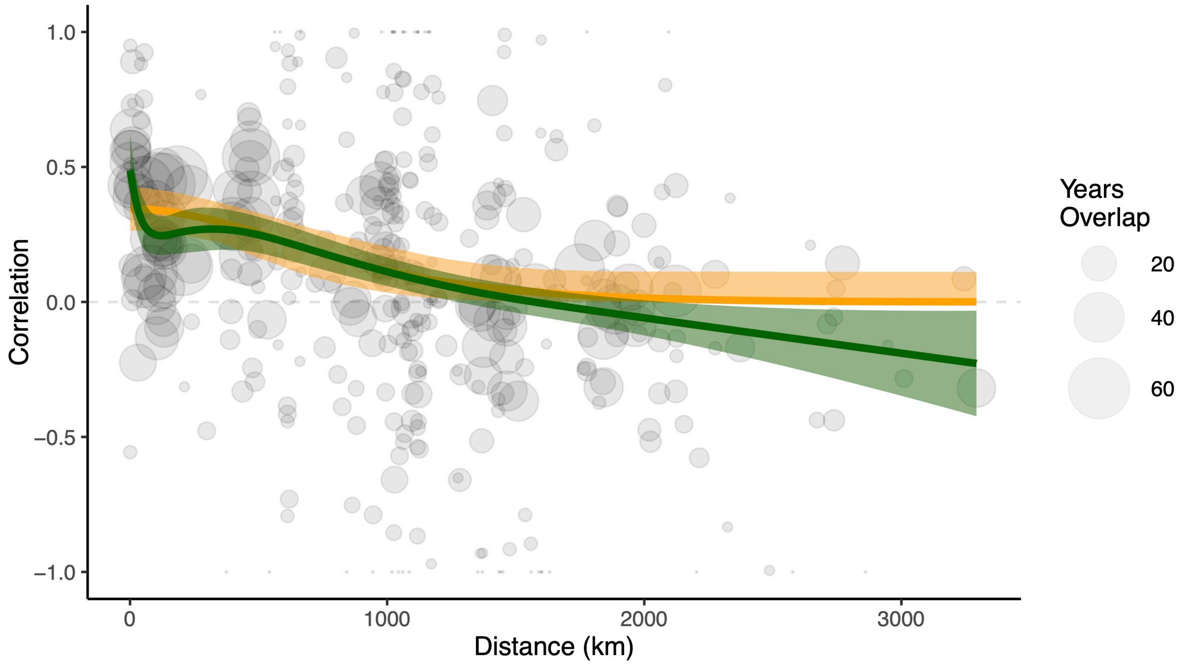

Figure S10 – Spatial synchrony of temporal variation in breeding demographic structure (proportion of subadults) in great tit populations 1956–2022. Distance between populations (km) is on the x-axis and correlation between paired sites is on the y-axis. The green line is the predicted correlation in temporal variation in subadult proportion between populations calculated using a semi-parametric approach, with light green shading representing 95% confidence intervals. This approach used a GAM that modelled the correlations in breeding demographic structure as a function of distance, allowing for on average negative synchrony. The orange line is the median estimate of spatial synchrony obtained from our main analytical approach (calculated in Equation 2) based on 2000 bootstrap replicates, with light orange shading representing 95% credible intervals. As can be seen, the estimated pattern of spatial synchrony is similar across both approaches at smaller spatial scales, with the addition of some evidence for synchrony becoming weakly negative at large distances through the semi-parametric approach.

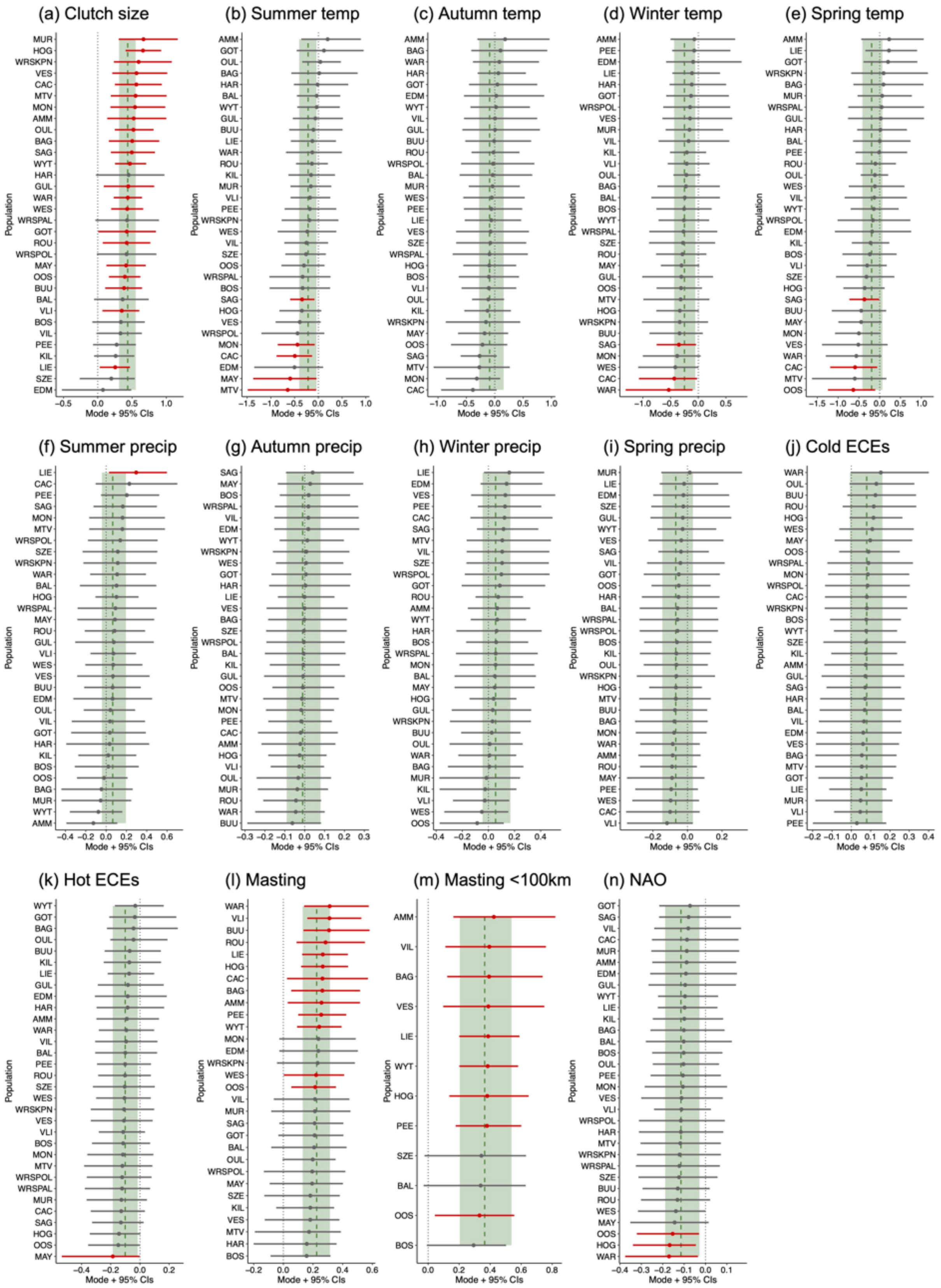

Figure S11 – Population-specific posterior means obtained from linear mixed-effects models which analyse the association between temporal variation in the proportion of breeding subadults and 14 reproductive and environmental variables (a–n). Each point corresponds to a specific population

(on the y-axis), and error bars denote 95% credible intervals. Red points and error bars denote significant effects where credible intervals do not overlap zero (black dashed line). Dotted green line and shading are the overall posterior mode and 95% credible intervals across all populations (as shown in the main text Figure 2).

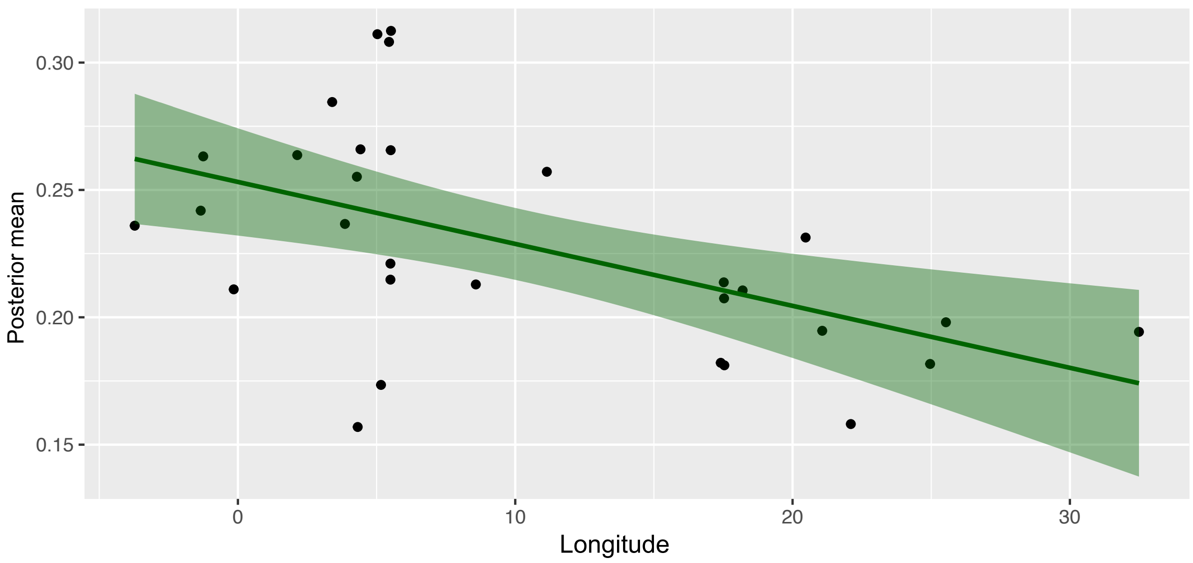

Figure S12 – Relationship between beech mast value and the proportion of breeding subadult great tits (posterior means from linear mixed-effect model) along the longitude of a population. The line is a linear regression which models the relationship between these two variables, and the shading around this shows the 95% confidence intervals. The relationship is significant and negative (Spearman's correlation: r = -0.517, p = 0.003).

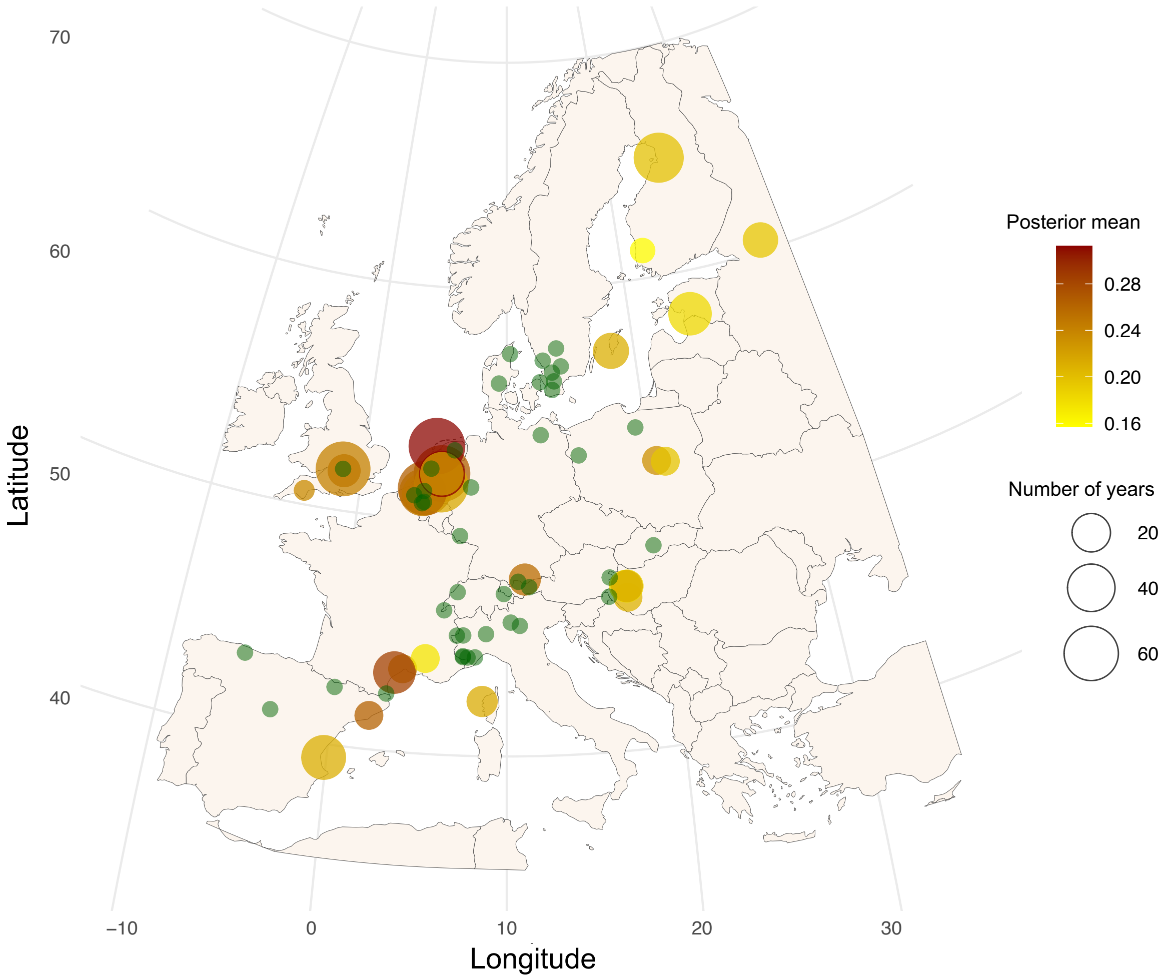

Figure S13 – Map of the 32 great tit study populations across Europe, with point size relative to the number of years in the time series and colour on a scale of yellow to dark red to indicate the strength of association between the proportion of breeding subadults and beech mast value (the magnitude of the posterior mean obtained from a linear mixed-effects model). The small green points refer to the locations at which mast data was collected and used in this analysis.

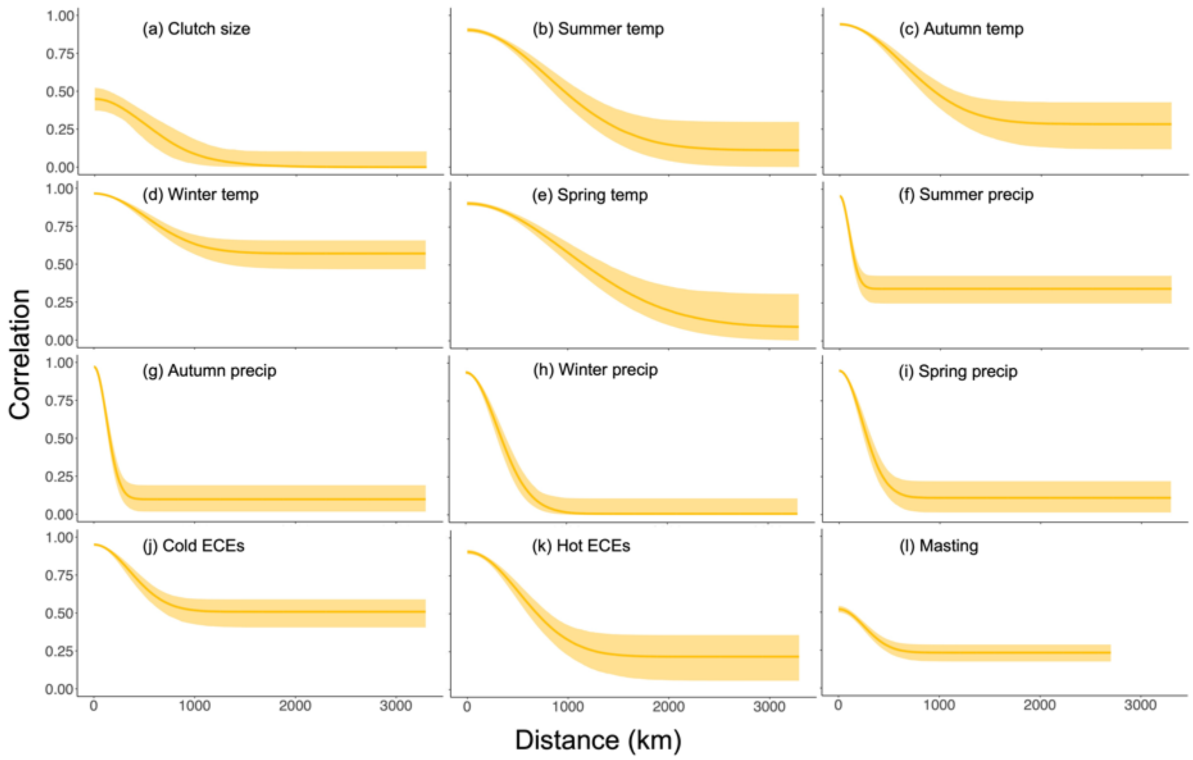

Figure S14 – Spatial synchrony of the reproductive and environmental variables in relation to distance between sites of data collection (i.e. the site of the 32 great tit populations for all variables except beech mast data). In all plots, distance between sites (km) is on the x-axis and correlation between paired sites is on the y-axis. The yellow line is the median estimate of spatial synchrony (calculated in Equation 2 in the main text) based on 2000 bootstrap replicates, with light yellow shading representing 95% credible intervals.

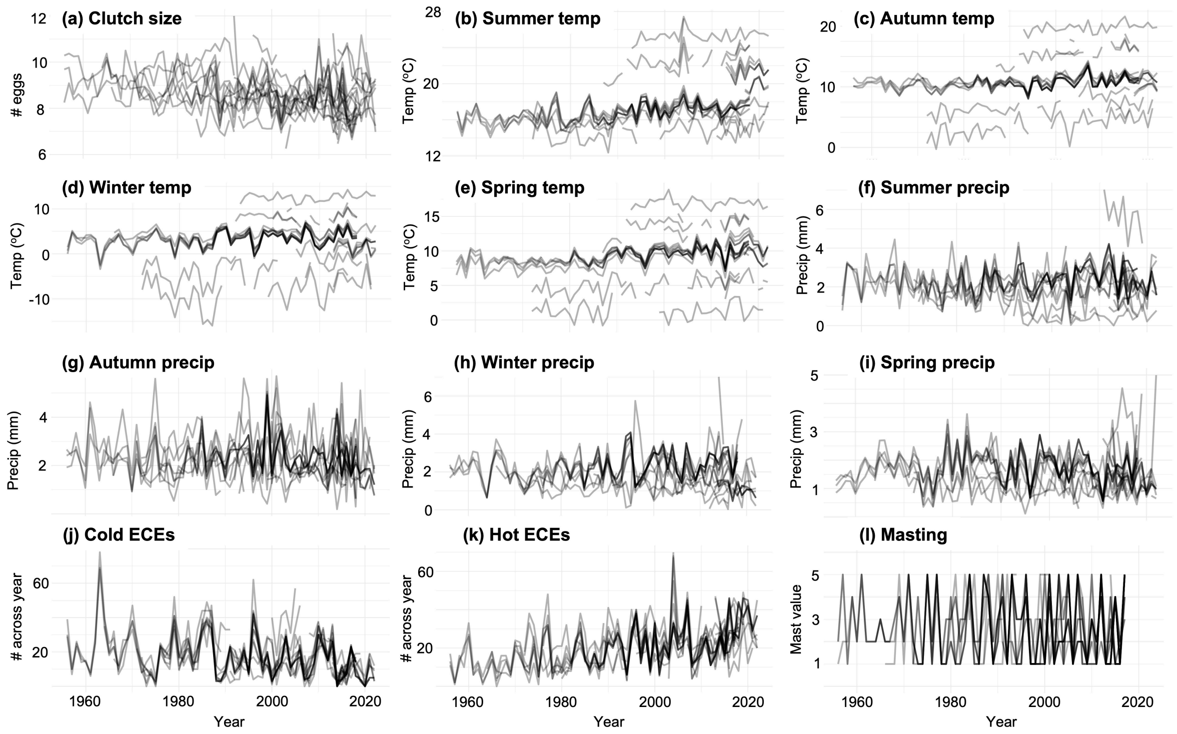

Figure S15 – Local temporal variation in the assessed reproductive and environmental variables for each population of great tits assessed in this study. Each line corresponds to a continuous time series for a single population.

References

Ahola, M.P., Laaksonen, T., Eeva, T. & Lehikoinen, E. (2009). Great tits lay increasingly smaller clutches than selected for: A study of climate- and density-related changes in reproductive traits. *Journal of Animal Ecology*, 78, 1298–1306.

Bailey, L.D. & van de Pol, M. (2016). Tackling extremes: Challenges for ecological and evolutionary research on extreme climatic events. *Journal of Animal Ecology*, 85, 85–96.

van Balen, J.H. (1980). Population fluctuations of the Great Tit and feeding conditions in winter. *Ardea*, 55, 143–164.

Bejer, B. & Rudemo, M. (1985). Fluctuations of Tits (Paridae) in Denmark and Their Relations to Winter Food and Climate. *Ornis Scandinavica*, 16, 29–37.

Bjørnstad, O.N., Ims, R.A. & Lambin, X. (1999). Spatial population dynamics: analyzing patterns and processes of population synchrony. *Trends Ecol Evol*, 14, 427–432.

Bogdziewicz, M., Hacket-Pain, A., Ascoli, D. & Szymkowiak, J. (2021). Environmental variation drives continental-scale synchrony of European beech reproduction. *Ecology*, 102, 1–10.

Bolte, A., Czajkowski, T. & Kompa, T. (2007). The north-eastern distribution range of European beech - a review. *Forestry*, 80, 413–429.

Bordjan, D. & Tome, D. (2014). Rain may have more influence than temperature on nest abandonment in the great tit parus major. *Ardea*, 102, 79–85.

Bouwhuis, S., Sheldon, B.C., Verhulst, S. & Charmantier, A. (2009). Great tits growing old: Selective disappearance and the partitioning of senescence to stages within the breeding cycle. *Proceedings of the Royal Society B: Biological Sciences*, 276, 2769–2777.

Boyce, M.S. & Perrins, C.M. (1987). *Optimizing Great Tit Clutch Size in a Fluctuating Environment*.

Clobert, J., Perrins, C.M., McCleery, R.H. & Gosler, A.G. (1988). Survival Rate in the Great Tit Parus major in Relation to Sex, Age, and Immigration Status. *Journal of Animal Ecology*, 57, 287–306.

Cornes, R.C., van der Schrier, G., van den Besselaar, E.J.M. & Jones, P.D. (2018). An Ensemble Version of the E-OBS Temperature and Precipitation Data Sets. *Journal of Geophysical Research: Atmospheres*, 123, 9391–9409.

Culina, A., Adriaensen, F., Bailey, L.D., Burgess, M.D., Charmantier, A., Cole, E.F., *et al.* (2021). Connecting the data landscape of long-term ecological studies: The SPI-Birds data hub. *Journal of Animal Ecology*, 90, 2147–2160.

Dhondt, A.A., Adriaensen, F., Matthysen, E. & Kempenaers, B. (1990). Nonadaptive clutch sizes in tits. *Nature*, 348, 723–725.

Engen, S., Lande, R., Seæther, B.-E. & Bregnballe, T. (2005). Estimating the pattern of synchrony in fluctuating populations. *Journal of Animal Ecology*, 74, 601–611.

Gamelon, M., Grøtan, V., Engen, S., Bjørkvoll, E., Visser, M.E. & Sæther, B.E. (2016). Density dependence in an age-structured population of great tits: Identifying the critical age classes. *Ecology*, 97, 2479–2490.

Gamelon, M., Vriend, S.J.G., Engen, S., Adriaensen, F., Dhondt, A.A., Evans, S.R., *et al.* (2019). Accounting for interspecific competition and age structure in demographic analyses of density dependence improves predictions of fluctuations in population size. *Ecol Lett*, 22, 797–806.

Gordo, O. & Sanz, J.J. (2010). Impact of climate change on plant phenology in Mediterranean ecosystems. *Glob Chang Biol*, 16, 1082–1106.

Gosler, Andrew. (1993). *The great tit*. *The great tit*, Hamlyn species guides. Hamlyn, London.

Greño, J.L., Belda, E.J. & Barba, E. (2007). Influence of temperatures during the nestling period on post-fledging survival of great tit Parus major in a Mediterranean habitat. *J Avian Biol*, 0, 071202183307007–0.

Hacket-Pain, A., Foest, J.J., Pearse, I.S., LaMontagne, J.M., Koenig, W.D., Vacchiano, G., *et al.* (2022). MASTREE+: Time-series of plant reproductive effort from six continents. *Glob Chang Biol*, 28, 3066–3082.

Herfindal, I., Tveraa, T., Stien, A., Solberg, E.J. & Grøtan, V. (2020). When does weather synchronize life-history traits? Spatiotemporal patterns in juvenile body mass of two ungulates. *Journal of Animal Ecology*, 89, 1419–1432.

Hurrell, J. & Phillips, A. (2003). *NAO Index Data provided by the Climate Analysis Section, NCAR, Boulder, USA, Hurrell*. Available at: https://climatedataguide.ucar.edu/climate-

data/hurrell-north-atlantic-oscillation-nao-index-station-based. Last accessed 25 March 2024.

Hurrell, J.W. (1995). Decadal trends in the North Atlantic oscillation: Regional temperatures and precipitation. *Science (1979)*, 269, 676–679.

Julliard, R., McCleery, R.H., Clobert, J. & Perrins, C.M. (1997). Phenotypic adjustment of clutch size due to nest predation in the Great Tit. *Ecology*, 78, 394–404.

Kelly, D. (1994). The evolutionary ecology of mast seeding. *Trends Ecol Evol*, 9, 465–470.

Kidd, L.R., Sheldon, B.C., Simmonds, E.G. & Cole, E.F. (2015). Who escapes detection? Quantifying the causes and consequences of sampling biases in a long-term field study. *Journal of Animal Ecology*, 84, 1520–1529.

Koenig, W.D. & Liebhold, A.M. (2016). Temporally increasing spatial synchrony of North American temperature and bird populations. *Nat Clim Chang*, 6, 614–617.

Lamb, P.J. & Peppler, R.A. (1987). North Atlantic oscillation: concept and an application. *Bull. Am. Meteorol. Soc.*, 68, 1218–1225.

Marrot, P., Garant, D. & Charmantier, A. (2017). Multiple extreme climatic events strengthen selection for earlier breeding in a wild passerine. *Philosophical Transactions of the Royal Society B: Biological Sciences*, 372.

Møller, A.P., Balbontín, J., Dhondt, A.A., Adriaensen, F., Artemyev, A., Bańbura, J., *et al.* (2020). Interaction of climate change with effects of conspecific and heterospecific density on reproduction. *Oikos*, 129, 1807–1819.

Moreno, J. & Møller, A.P. (2011). Extreme climatic events in relation to global change and their impact on life histories. *Curr Zool*, 57, 375–389.

Perrins, C.M. (1965). Population Fluctuations and Clutch-Size in the Great Tit, Parus major L. *Journal of Animal Ecology*, 34, 601–647.

Perrins, C.M. (1979). *British tits*. 1st edn. Collins, London.

Perrins, C.M. & Moss, D. (1975). Reproductive Rates in the Great Tit. *Journal of Animal Ecology*, 44, 695–706.

Pettifor, R.A., Perrins, C.M. & McCleery, R.H. (2001). The individual optimization of fitness: variation in reproductive output, including clutch size, mean nestling mass and offspring recruitment, in manipulated broods of great tits Parus major . *Journal of Animal Ecology*, 70, 62–79.

Post, E. & Stenseth, N.C. (1999). Climatic variability, plant phenology, and northern ungulates. *Ecology*, 80, 1322–1339.

Regan, C.E. & Sheldon, B.C. (2023). Phenotypic plasticity increases exposure to extreme climatic events that reduce individual fitness. *Glob Chang Biol*, 29, 2968–2980.

Sæther, B.-E., Engen, S., Grøtan, V., Fiedler, W., Matthysen, E., Visser, M.E., *et al.* (2007). The extended Moran effect and large-scale synchronous fluctuations in the size of great tit and blue tit populations. *Journal of Animal Ecology*, 76, 315–325.

Schneider, D.P., Deser, C., Fasullo, J. & Trenberth, K.E. (2013). Climate data guide spurs discovery and understanding. *Eos, Transactions American Geophysical Union*, 94, 121–122.

Silvertown, J.W. (1980). The evolutionary ecology of mast seeding in trees. *Biological Journal of the Linnean Society*, 14, 235–250.

Sullivan, B.L., Wood, C.L., Iliff, M.J., Bonney, R.E., Fink, D. & Kelling, S. (2009). eBird: A citizen-based bird observation network in the biological sciences. *Biol Conserv*, 142, 2282–2292.

Svensson, L. (1992). *Identification guide to European passerines*. 4th, revd. edn. Lars Svensson ; British Trust for Ornithology, Stockholm : Thetford.

Vacchiano, G., Hacket-Pain, A., Turco, M., Motta, R., Maringer, J., Conedera, M., *et al.* (2017). Spatial patterns and broad-scale weather cues of beech mast seeding in Europe. *New Phytologist*, 215, 595–608.

Vriend, S.J.G., Grøtan, V., Gamelon, M., Adriaensen, F., Ahola, M.P., Álvarez, E., *et al.* (2023). Temperature synchronizes temporal variation in laying dates across European hole-nesting passerines. *Ecology*, 104.

Wanner, H., Brönnimann, S., Casty, C., Gyalistras, D., Luterbacher, J., Schmutz, C., *et al.* (2001). North Atlantic Oscillation - concepts and studies. *Surv Geophys*, 22, 321–382.
